# Distinctive Gene Expression Patterns Define Endodormancy to Ecodormancy Transition in Apricot and Peach

**DOI:** 10.3389/fpls.2020.00180

**Published:** 2020-02-28

**Authors:** Jiali Yu, Anna O. Conrad, Véronique Decroocq, Tetyana Zhebentyayeva, Daniel E. Williams, Dennis Bennett, Guillaume Roch, Jean-Marc Audergon, Christopher Dardick, Zongrang Liu, Albert G. Abbott, Margaret E. Staton

**Affiliations:** ^1^ Genome Science and Technology Program, University of Tennessee, Knoxville, TN, United States; ^2^ Forest Health Research and Education Center, University of Kentucky, Lexington, KY, United States; ^3^ Department of Plant Pathology, The Ohio State University, Columbus, OH, United States; ^4^ UMR 1332 Biologie du Fruit et Pathologie, Equipe de Virologie, INRA, Universite de Bordeaux, Villenave d'Ornon, France; ^5^ Department of Ecosystem Science and Management, Schatz Center for Tree Molecular Genetics, the Pennsylvania State University, University Park, PA, United States; ^6^ Center for Environmental Biotechnology, University of Tennessee, Knoxville, TN, United States; ^7^ Appalachian Fruit Research Station, United States Department of Agriculture—Agriculture Research Service, Kearneysville, WV, United States; ^8^ GAFL Fruit and Vegetable Genetics and Breeding, INRA Centre PACA, Montfavet, France; ^9^ Department of Entomology and Plant Pathology, Institute of Agriculture, University of Tennessee, Knoxville, TN, United States

**Keywords:** dormancy, *Prunus*, floral buds, transcriptome, chill requirement, bloom date, co-expression network, RNASeq

## Abstract

Dormancy is a physiological state that plants enter for winter hardiness. Environmental-induced dormancy onset and release in temperate perennials coordinate growth cessation and resumption, but how the entire process, especially chilling-dependent dormancy release and flowering, is regulated remains largely unclear. We utilized the transcriptome profiles of floral buds from fall to spring in apricot (*Prunus armeniaca*) genotypes with contrasting bloom dates and peach (*Prunus persica*) genotypes with contrasting chilling requirements (CR) to explore the genetic regulation of bud dormancy. We identified distinct gene expression programming patterns in endodormancy and ecodormancy that reproducibly occur between different genotypes and species. During the transition from endo- to eco-dormancy, 1,367 and 2,102 genes changed in expression in apricot and peach, respectively. Over 600 differentially expressed genes were shared in peach and apricot, including three DORMANCY ASSOCIATED MADS-box (*DAM*) genes *(DAM4*, *DAM5*, and *DAM6*). Of the shared genes, 99 are located within peach CR quantitative trait loci, suggesting these genes as candidates for dormancy regulation. Co-expression and functional analyses revealed that distinctive metabolic processes distinguish dormancy stages, with genes expressed during endodormancy involved in chromatin remodeling and reproduction, while the genes induced at ecodormancy were mainly related to pollen development and cell wall biosynthesis. Gene expression analyses between two *Prunus* species highlighted the conserved transcriptional control of physiological activities in endodormancy and ecodormancy and revealed genes that may be involved in the transition between the two stages.

## Introduction

In temperate regions, many perennial plants protect their buds and reproductive tissues from winter cold by entering a state of dormancy. To transit from dormancy to bud-burst, these trees must be exposed to a particular period of chilling temperatures and warm temperatures, referred to as chilling requirements (CR) and heat requirements (HR) respectively. The CR for bud break is an intrinsic part of temperate tree phenology that varies between and within species based on adaptations to the climate of their native range (Luedeling, 2012). Various models exist for calculating CR, but they are usually measured as the number of accumulated hours in a specific range, around 0–7°C (Richardson, 1974; Erez, 1979; Shaltout, 1983; Hänninen, 1990; Cesaraccio et al., 2004). For agricultural tree crops, cultivars must be carefully selected for each growing region in order to provide a sufficient amount of cold for regular flowering and reliable fruit or nut production (Luedeling and Brown, 2011). With global climate change, understanding dormancy and its control are critical for the productivity of fruit trees, as increasing or irregular seasonal temperatures impact optimal timing for important phenological traits (Hatfield and Prueger, 2015). Lack of fulfillment of CR due to warm winters affects bud break, resulting in low flowering rates and thus low fruit yield as the fruit trees do not fulfill their CR (Viti and Monteleone, 1991). In addition, early blooming trees can suffer significant yield loss from frost damage to floral blooms during unusually late periods of freezing in the spring (Rieger, 1989).

Winter dormancy is divided into two stages, endodormancy and ecodormancy (Lang et al., 1987). Floral bud endodormancy is a state defined by physiological inhibition of flowering and is induced by cold temperatures and/or short photoperiods (Horvath et al., 2003; Foley et al., 2009). Fulfillment of CR marks the transition point from endodormancy to ecodormancy. In ecodormancy, buds regain competency to respond to external environmental factors. They remain ecodormant under unfavorable growth conditions (e.g. cold temperatures), but quickly progress toward budburst when favorable conditions are present. Plants that do not receive sufficient chilling fail to transition to ecodormancy, which leads to failing to bloom or blooming erratically in the spring (Luedeling et al., 2011). The transition from endodormancy to ecodormancy is irreversible, however, there is not a phenotype which uniquely identifies the physiological state of endodormancy other than measurements of chill accumulation and more recently, studies of starch accumulation in sweet cherry floral buds (Anderson et al., 1985; Citadin et al., 2001; Chavarria et al., 2009; Fadón et al., 2018a). More studies linking floral bud endodormancy with specific physiological networks are necessary to determine what drives the transition from endo- to eco-dormancy and how this transition is regulated at both the genetic and physiological levels.

*Prunus* spp. have genotypes exhibiting CRs ranging from approximately 100 hours to over 1,000 hours (Audergon, 1993; Caruso et al., 1997; Valentini et al., 2002; Alburquerque et al., 2008; Dirlewanger et al., 2012; Quero-Garcia et al., 2016). Genotypes with a broad phenotypic range for this trait provide excellent materials for the study of genetic control of CR and molecular activities at different stages of dormancy. Previous genetic studies on dormancy associated traits suggest that CR is a complex quantitative trait and has a major effect on flowering time in *Prunus* spp. including almond (*P. dulcis*) (Sánchez-Pérez et al., 2012), apricot (*P. armeniaca*) (Olukolu et al., 2009), peach (*P. persica*) (Fan et al., 2010; Zhebentyayeva et al., 2014), and sweet cherry (*P. avium*) (Castède et al., 2014). Some major quantitative trait loci (QTL) for CR appear to overlap orthologous genomic regions across species, suggesting shared underlying molecular mechanisms (Dirlewanger et al., 2012). For example, the most significant QTL responsible for CR and flowering time was identified on LG4 in sweet cherry (Castède et al., 2014) and almond (Sánchez-Pérez et al., 2012). Despite this extensive research, the only QTL region for CR and blooming date (BD) with strongly supported candidate genes is a major QTL on LG1 from peach (Fan et al., 2010; Zhebentyayeva et al., 2014). The strong association between this QTL region on chromosome 1 and CR was also identified from a genome-wide association study on over 400 peach genotypes (Li et al., 2019). This region contains six tandemly repeated DORMANCY ASSOCIATED MADS-box (*DAM1-6*) transcription factors, previously mapped to the *evg (evergrowing)* locus (Wang et al., 2002; Bielenberg et al., 2004). The deletion of four of the six *DAM* genes is associated with the *evergrowing* phenotype in peach, which exhibits an inability to cease growth in winter (Bielenberg et al., 2008). Gene profiling reveals *DAM1–4* peak in expression during bud set and may influence dormancy onset (Li et al., 2009; Yamane et al., 2011) while levels of *DAM5* and *DAM6* are high at the beginning of endodormancy and decreased steadily during chilling period (Li et al., 2009; Jiménez et al., 2010b; Yamane et al., 2011; Leida et al., 2012a). This pattern suggests that these genes likely play a significant role in maintaining the endodormant state. Recent studies of *DAM* genes and their homologs *SHORT VEGETATIVE PHASE-like* (*SVL*) genes suggest they are cooperatively regulated by several transcription factors, hormones, and epigenetic factors (da Silveira Falavigna et al., 2018). Wang et al. (2019) propose peach *TEOSINTE BRANCHED1* transcription factor negatively regulates DAM5 and DAM6 expression, resulting in dormancy release (Wang et al., 2019). Leida et al. (2012b) reported that histone modifications in *DAM6* promoter, the second exon and the second intron are involved in dormancy release (Leida et al., 2012b). Sequencing of the *DAM* gene region in selected members of a QTL population of peach revealed that large intronic insertions in *DAM5* and *DAM6* are associated with the low chill phenotype, and Zhebentyayeva et al. (2014) hypothesize these insertions change the epigenetic factors of the locus to influence expression (Zhebentyayeva et al., 2014).

In *Prunus*, winter buds were originally considered to be in a resting state and thus this state was termed “dormancy.” However, both endodormant and ecodormant *Prunus* buds are now known to have unique and changing transcriptional profiles, continued metabolic activities including starch accumulation and hormone fluctuations, and ongoing floral structure differentiation (Reinoso et al., 2002; Zhu et al., 2015; Chmielewski et al., 2017; Zhang et al., 2018). This differs from other species such as pear, which have a true resting state at endodormancy (Saito et al., 2015b). Previous studies have profiled expression for a subset of genes during bud dormancy in peach (*Prunus persica*) using suppression subtractive hybridization (Leida et al., 2010) and in apricot (*Prunus armeniaca*) using cDNA amplified fragment length polymorphism (Čechová et al., 2012). However, no high throughput global gene expression studies are available in either species. In Japanese apricot (*Prunus mume*), a more recent transcriptome study has profiled global gene expression patterns during bud dormancy (Zhang et al., 2018). Transcriptomic and metabolic profiles indicate that gibberellins (GA) and abscisic acid (ABA) likely play a role in bud dormancy (Zhang et al., 2018). Based on their data and previous results from others, Zhang et al. (2018) proposed a molecular model of dormancy control: cold temperatures induce c-repeat binding factor (*CBF*) transcripts, which promote the expression of *DAM* genes and inhibit the GA signaling pathway, resulting in endodormancy. The long-term cold period then reduces *CBF* and *DAM* gene expression, leading to dormancy release (Zhang et al., 2018). However, Japanese apricot floral timing, unlike other *Prunus* species, are more strongly correlated to HR rather than CR (Kitamura et al., 2017). Research from a wide variety of tree species has yielded other proposed mechanisms for dormancy regulation, including epigenetic regulation of key gene networks through DNA and histone methylation (Lloret et al., 2018). Studies in poplar also support that hormones such as ABA participate in the establishment and release of dormancy (Rinne et al., 2011; Singh et al., 2018; Tylewicz et al., 2018). Low temperature and short photoperiod induce poplar bud dormancy by upregulating ABA signaling, which increases poplar *SVL* expression (Singh et al., 2018; Singh et al., 2019). *SVL* is likely involved in dormancy initiation and bud break (Busov, 2019). ABA has been reported to induce dormancy by increasing the frequency of plasmodesmata closure, which inhibits growth signal transport to the apical meristem (Tylewicz et al., 2018). Overexpression of *Prunus mume DAM6* in apple delays bud break by increasing ABA and decreasing cytokinin levels (Yamane et al., 2019). While ethylene has mainly been characterized as a phytohormone controlling dormancy onset in trees such as poplar and birch (Ruonala et al., 2006; Ruttink et al., 2007), it also may be important for later stages as exogenous application can delay budbreak (Liu and Sherif, 2019).

This study examined global gene expression profiles over dormancy states in the contrasting CR and BD of peach and apricot genotypes, allowing us to differentiate candidate genes responsible for endodormancy maintenance and/or endodormancy to ecodormancy transition. Species of *Prunus* including peach (*P. persica*), apricot (*P. armeniaca*), and plum (*P*. *domestica*) have high genomic synteny (Dirlewanger et al., 2004; Jung et al., 2009). Based on extensive genomic data, the current study proposes to characterize shared molecular mechanisms during the endodormancy to ecodormancy transition in floral bud tissues across two related *Prunus* species, peach and apricot. RNASeq analysis was performed to explore the transcriptomic changes as dormancy progressed in floral buds from four apricot genotypes with contrasting BD, along with floral buds from four peach genotypes with contrasting CR phenotypes. After integrating the peach and apricot RNASeq data, shared differential expression genes between endodormancy and ecodormancy stages were identified. Moreover, co-expression networks revealed similar biological pathways triggered by endodormancy or ecodormancy in both species. Therefore, examination of gene expression patterns in different *Prunus* species is expected to shed light on the physiological and molecular similarities of dormancy across species and potentially help to generate new candidate genes for previously identified QTLs controlling CR.

## Materials and Methods

### Plant Materials

Five apricot genotypes grown at INRA-PACA Domaine des Pins de l'Amarine, France were used for this study: A1956 (Palsteyn, n=2), A2137 (Bakour, n=3), A1267 (Badami, n=3), A660 (Bergeron, n=3), and A2312 (Flamingold, n=1). Two late blooming genotypes (A660, A1267) and three early blooming genotypes (A1956, A2137, A2312) were selected, with the historical record of bloom dates reported in Conrad et al. (Conrad et al., 2019). While CR has not been directly measured for these genotypes, the bloom date (BD) phenotype is correlated with CR (Fan et al., 2010): early BD genotypes have low CR while late BD genotypes have high CR. Floral buds from clonally propagated (grafted) trees of each genotype were collected at 0, 100, 400, and 800 chill hours regardless of their developmental stages, starting from October 29th, 2015. Two additional collections were made at specific developmental stages, when sepals became visible (C2) and when petals became visible (D1) (Conrad et al., 2019).

Peach genotypes A209 (300 CR), A340 (300 CR), A318 (850 CR), and A323 (1,100 CR) were selected from an F_2_ population derived from low CR cultivar “Fla.92-2C” by high CR cultivar “Contender” (Fan et al., 2010; Zhebentyayeva et al., 2014) located at Clemson University. Floral buds from four clones of each genotype were collected at 0, 100, 600, and 1,000 chill hours and the stage of pre-bloom, from October 14th, 2015 until March 17th, 2016 (**Table 1**). No collection was made at the 1,000 chill hours time point for the low chill genotypes as the buds had already flowered.

**Table 1:** Sampling time points and chilling requirements (CRs) for *Prunus persica* trees.

Phenotype Genotype	Low-CR		High-CR	
	A209	A340	A318	A323
Chill requirement^1^ (hours)	300	300	850	1,100
Time point	Sampling date			
0 CH^2^	14/16-Oct-15	14/16-Oct-15	14/16-Oct-15	14/16-Oct-15
100 CH	23-Nov-15	23-Nov-15	23-Nov-15	23-Nov-15
600 CH	28-Jan-16	28-Jan-16	28-Jan-16	28-Jan-16
1,000 CH^3^	NS	NS	27-Feb-16	27-Feb-16
Pre-bloom (PB)	10-Feb-16	10-Feb-16	12-Mar-16	17-Mar-16

^1^CR reported in (Zhebentyayeva et al., 2014.)

^2^Samples were collected over a 2 d period, from October 14th to 16th, 2015.

^3^NS, not sampled.

### RNA Extraction and Sequencing

RNA was extracted from floral buds of five apricot genotypes (four as specified below for RNASeq and one genotype A2312 for qPCR) as previously described (Conrad et al., 2019). Not all samples yielded high quality RNA. Of the 24 genotype by time point collections, 14 had successful extractions of all three biological replicates, eight had successful extractions of only two biological replicates, and two had only a single biological replicate (A2137 at 0 chill hours and A1267 at 0 chill hours) (**Supplementary Tables 1** and **2**). The 60 RNA samples from four apricot genotypes (A1267, A660, A1956, A2137) were submitted to MOgene, LLC (St. Louis, MO, USA) for library preparation and sequencing by an Illumina NextSeq 500, yielding 50 bp single-end reads.

Peach RNA samples were prepared by grinding 1 g of flower buds to a fine powder in the presence of liquid nitrogen followed immediately by the addition of 5 ml cold Invitrogen Plant RNA Reagent (Thermo Fisher Scientific, Waltham, MA, Cat# 12322-012), aliquoted 1 ml into microcentrifuge tubes and following the manufacturer's instructions. RNA pellet was dissolved in 100 μl of RNASecure (Thermo Fisher Scientific, Waltham, MA, Cat# AM7006). Additional removal of DNA was required by adding an equal volume Sigma Tri-Reagent (Sigma, St. Louis, MO, Cat# T9424) using manufacturer's instructions with two additional purification steps using Phenol : Chloroform and final elution in 100 μl of RNASecure (Thermo Fisher Scientific, Waltham, MA, Cat# AM7006). One μg of RNA sample were loaded on a Low EEO/Multipurpose Agarose gel (Thermo Fisher Scientific, Waltham, MA, Cat# BP160) and normalized using a Typhoon 9600 FLS (GE Healthcare, Marlborough, MA) with ImageQuant TL Image Analysis Software version 8.1 (GE Healthcare, Marlborough, MA). All genotype by time point combinations had at least three biological replicates and most had four biological replicates (**Supplementary Tables 1** and **2**). Sixty-nine RNA samples were submitted to GeneWiz (South Plainfield, NH, USA) for library preparation and sequencing on the HiSeq platform to generate 150 bp paired-end reads.

### Quality Control

RNA-seq yielded approximately 19–35 million reads per library. Raw reads were analyzed for quality by FastQC (Andrews, 2010). Adaptors and low-quality reads were trimmed by skewer (Jiang et al., 2014).

### Read Alignment and Gene Quantification

As an apricot reference genome is not available, and peach and apricot are closely related, the peach reference genome was used as a reference for both species. Initial read alignments revealed the peach RNAseq libraries contained significant portions of reads derived from rRNA. rRNA filtering was conducted before re-aligning to reference genome. Raw reads were mapped to small and large subunit rRNAs from SILVA ribosomal RNA database (Quast et al., 2013) and the mapped reads were removed. STAR (Dobin et al., 2013) was used to align the clean apricot and peach RNA-seq reads to both *Prunus persica* genomes: *Prunus persica* v1.0 (The International Peach Genome Initiative et al., 2013) and *Prunus persica* v2.0 (Verde et al., 2017). The aligned reads were analyzed by HTSeq-count (Anders, Pyl et al., 2015), which counts for the aligned reads mapped to the exons. The *Prunus persica* v2.0 was used for all analyses except for the DAM region, which is correctly annotated in the original version of the genome *Prunus persica* v1.0 (**Supplementary Figure 1**). For the HTSeq-count results, the DAM region in *Prunus persica* v2.0 (from Prupe.1G531100 to Prupe.1G531700, peach chromosome Pp01 from 43,417,246 to 43,480,648 bases) was removed and replaced by the read counts from the six *DAM* genes (ppa018667m, ppb017585m, ppa010758m, ppa011123m, ppa010822m, and ppa010714m) annotated in *Prunus persica* v1.0 assembly.

### Differential Expression Analysis and Functional Enrichment

Gene counts were analyzed for gene-level differential expression using DESeq2 (Love et al., 2014). Sample distances were calculated using the regularized log transformation of gene counts available from the DESeq2 package. The principal component (PC) analysis used the first and second largest sample distances as the first PC and second PC. Two Wald tests were performed. The first utilized one factor, stage of development. A second test was run with one factor, phenotype, coded as either early bloom or late bloom for each sample. Significantly differentially expressed genes (DEGs) were identified with fold change > 2 and adjusted p-value < 0.05. Genes of interest were annotated with gene ontology (GO) by AgriGO v2.0 (Tian et al., 2017) using both peach *Prunus persica* v2.0 and *Arabidopsis* TAIR10 genomes. The *Arabidopsis* genes corresponding to peach genes were obtained as the best hit of *Arabidopsis* as recorded in the annotation information file from Phytozome12 (Goodstein et al., 2012). GO enrichment analysis was conducted using the hypergeometric statistical model and p-values were adjusted by the Hochberg method. The GO terms with FDR < 0.05 were regarded as significantly enriched.

### Differential Co-Expression Analysis and Network Enrichment Analysis

Weighted Gene Correlation Network Analysis (WGCNA) (Zhang and Horvath, 2005; Langfelder and Horvath, 2008) was used to identify modules of highly correlated genes based on the DESeq2 normalized gene expression data following the steps in the documentation for the WGCNA Package (Langfelder and Horvath, 2014). Genes with 0 normalized count were removed. The expression profiles of the remaining genes were hierarchically clustered into modules containing at least 100 genes. The expression profiles of each module were summarized using the first PC as the module eigengene (ME). Module-traits correlations were calculated by Pearson correlation coefficient (PCC) where each module was represented by its ME, and each developmental stage was represented with a numeric vector with “1” for the trait, and “0” for all the others. Genes from selected modules were annotated with the *Arabidopsis* gene as described above. The GO enrichment networks were analyzed by BiNGO in Cytoscape (Maere et al., 2005).

### Reverse Transcription Quantitative PCR (RT-qPCR)

Total RNA was extracted as previously described (Conrad et al., 2019). One RNA sample from each of three apricot genotypes (A2137, A1956, A2312) at time points 100, 400, 800, and sepal was subjected to real-time qPCR analysis using the Luna^®^ Universal one step RT-qPCR kit (E3005S, New England Biolabs Inc, MA) with the primers listed in **Table 2**. Expression levels of mRNAs of interest were normalized for the 18S RNA levels. Three technical replicates were used for each sample.

**Table 2:** Primers used for qPCR.

Target transcript	Primer sequence (5' to 3')
18S_F	GTTACTTTTAGGACTCCGCC
18S_R	TTCCTTTAAGTTTCAGCCTTG
DAM6_F	TACTGGACCTGCGTTTGTGGAGCC
DAM6_R	TGTTGCAGCTGGTGGAGGTGGCAATT
Prupe.1G104900_F	TCATCTTCCGCTGCCTTTGTAGCCT
Prupe.1G104900_R	GACACTGCCAAGAACACCAAGGACA
Prupe.2G122600_F	GGAGAAATTGGAACGCCTGTGC
Prupe.2G122600_R	TGAGCCCTCAGTTGCTAGTTCAG
Prupe.5G014900_F	TCCCTTTGGACAGATTCCAGTGC
Prupe.5G014900_R	GCAGCCTCTTTCAGGTTGTTGTG
Prupe.7G084900_F	ACCATTTGCCGGATGGATGGAAG
Prupe.7G084900_R	CAACCATGTCAGCTGGAACCAC

### Floral Bud Dissection and Imaging

Ten flower buds were randomly selected from each of the four peach genotypes and dissected longitudinal, slightly off-center using razor blades (Electron Microscopy Sciences Stainless Steel “PTFE” coated Double Edge, PERSONNA brand.004” thick, Fisher Scientific, Waltham, MA, USA, Cat# 50-949-411). The dissected buds were imaged using Nikon SMZ1500 Stereoscopic Zoom Microscope (Nikon Instruments Inc, Melville, NY, USA) with a Pax-it! ARC Model PS-CM camera (Villa Park, IL, USA).

### Multivariate Analysis of Variance

Permutational multivariate analysis of variance (PERMANOVA) (Anderson, 2001; Anderson, 2017) of peach samples was analyzed by “*adonis”* function from R package “vegan” (Oksanen et al., 2019). The regularized log-transformed gene counts were used for the PERMANOVA and the ratios of rRNA content, genotypes, and time points were used as factors.

## Results

### Quantification of Gene Expression During Dormancy in Apricot

In *Prunus* spp., individual genotypes have specific CR accommodating various climatic zones, with trees with lower CR blooming earlier on average and trees with higher CR blooming later (Alburquerque et al., 2008; Fan et al., 2010; Castède et al., 2014; Benmoussa et al., 2017; Fadón et al., 2018b; Balogh et al., 2019). For this experiment, genotypes with varying average bloom dates when planted in the same location were selected. To determine the phenotypes of the four apricot genotypes, the bloom dates from 1999 to 2013 were recorded. Genotypes A1956 and A2137 reached 50% bloom after an average of 60 Julian calendar days and are hereafter referred to as “early blooming” (Audergon, 1993; Dirlewanger et al., 2012; Andreini et al., 2014; Quero-Garcia et al., 2016). Genotypes A660 and A1267 reached 50% bloom after more than 70 Julian days on average (“late blooming”) (**Supplementary Figure 2A**). A2137 was the earliest genotype entering sepal visible stage at 577 chill hours, while the latest one to enter the visible sepal stage at 1,002 chill hours was genotype A1267 (**Supplementary Figure 2B**).

Over the course of a single winter, bud samples were collected at 0, 100, 400, and 800 chill hours as well as at the sepal visible stage and the petal visible stage. Due to the variation in dormancy release time, early blooming trees had already bloomed at 800 chill hours while the late blooming trees were still dormant. Therefore, samples from A660 and A1267 at 800 chill hours are labeled as Bud-800, and samples from A2137 and A1956 are labeled as Flower-800 for clarity in developmental stage of tissues collected.

RNA sequencing was performed with individual libraries for each set of buds collected from each individual tree, yielding from 19.1 million to 35.2 million raw reads per library. Peach and apricot have structurally similar genomes as identified by the comparison of apricot and peach genetic maps (Dirlewanger et al., 2004; Arús et al., 2006). As a *Prunus armeniaca* reference genome and annotations are not publicly available, the peach genome was tested as a possible alternative. Over 90% of the raw reads were successfully mapped, and approximately 70%–80% of the reads map to annotated genes (**Supplementary Figure 3**). All additional apricot results are reported in reference to the *Prunus persica* v2.0 genome.

**Figure 1:**
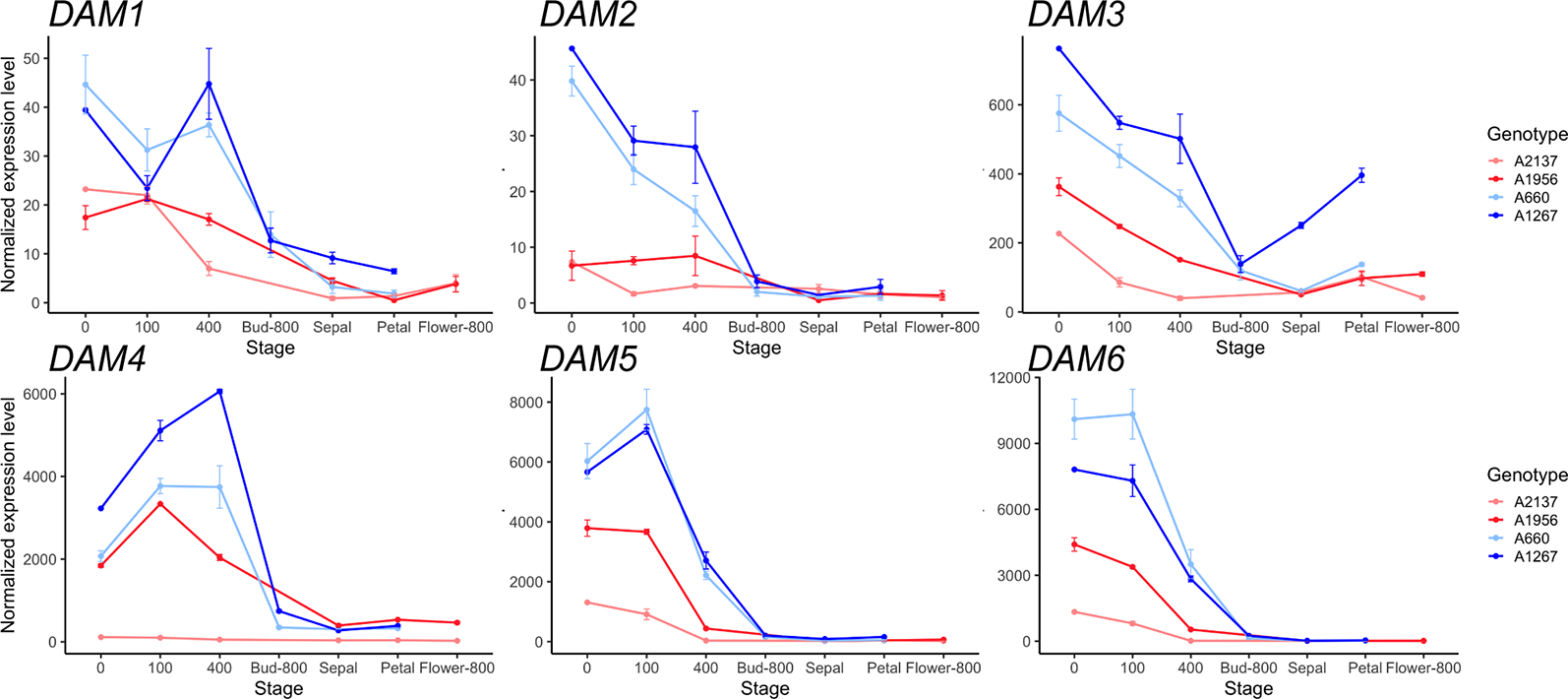
The expression of six *DAM* genes during dormancy through bud break in the four apricot genotypes. Top: *DAM1* to *DAM3*, Bottom: *DAM4* to *DAM6*. Two red lines represent early blooming genotypes, two blue lines represent late blooming genotypes (mean ± SE). Biological replicates range from 1 to 3 for each genotype and time point (**Supplementary Tables 1** and **2**).

The expression of the *DAM* genes, a tandem duplication of six genes that are well-known regulators of dormancy in peach, were examined. The six *DAM* genes were found to be misannotated in *Prunus persica* v2.0 genome, with exons from *DAM* 1–3 grouped together in a single gene model (**Supplementary Figure 1**). Therefore, we replaced the gene counts in this region using the *Prunus persica* whole genome assembly v1.0, where the *DAM* genes have been manually annotated (The International Peach Genome Initiative et al., 2013). The six *DAM* genes had lower expression levels in the early blooming genotypes than in the late blooming genotypes throughout dormancy to bloom (**Figure 1**). *DAM1* to *DAM3* genes had relatively high expression levels at the beginning of chilling, and were downregulated as chill hours increased. *DAM1* in the late blooming genotypes was increased at 400 chill hours, while *DAM1* expression was downregulated at 400 chill hours in early blooming genotypes (**Figure 1**). Notably, *DAM4* and *DAM5* in the late blooming genotypes were highly expressed at the beginning of endodormancy, and downregulated after 100 chill hours (i.e. *DAM5*) or 400 chill hours (i.e. *DAM4*). In the early blooming genotype A2137, the expression levels of *DAM4* had little change during dormancy. *DAM6* expression levels were decreased consistently in all genotypes as chill hours accumulated. However, the four genotypes have different expression levels at 0 chill hour: the earliest blooming genotype A2137 had the lowest *DAM6* expression while the late blooming genotype A660 had the highest expression level (**Figure 1**). Overall, the expression of the six *DAM* genes were downregulated at 400 chill hours in the early blooming genotypes, while in the late blooming genotypes, they reached the same level at 800 chill hours (**Figure 1**). This suggests the expression level is correlated with the timing of growth resumption. The *DAM* genes are highly expressed at the onset of dormancy followed by downregulation at bud break. This is consistent with previous studies that *DAM* genes are associated with dormancy regulation (Bielenberg et al., 2008; Li et al., 2009; Jiménez et al., 2010b). Similar gene expression profiles of *DAM5* and *DAM6* were observed in other apricot genotypes from the same year (Balogh et al., 2019). The difference of *DAM5* expression levels in different apricot genotypes at 400 chill hours was also seen in different peach genotypes (Leida et al., 2012c). However, our results for *DAM5* and *DAM6* expression levels differ from the findings of Li et al. (2009) who reported that *DAM5* and *DAM6* were upregulated until CR was met in peach (Li et al., 2009).

### Transcriptome Profiles Clustered by Bud Developmental Stages, Regardless of Chill Hours

**Figure 2:**
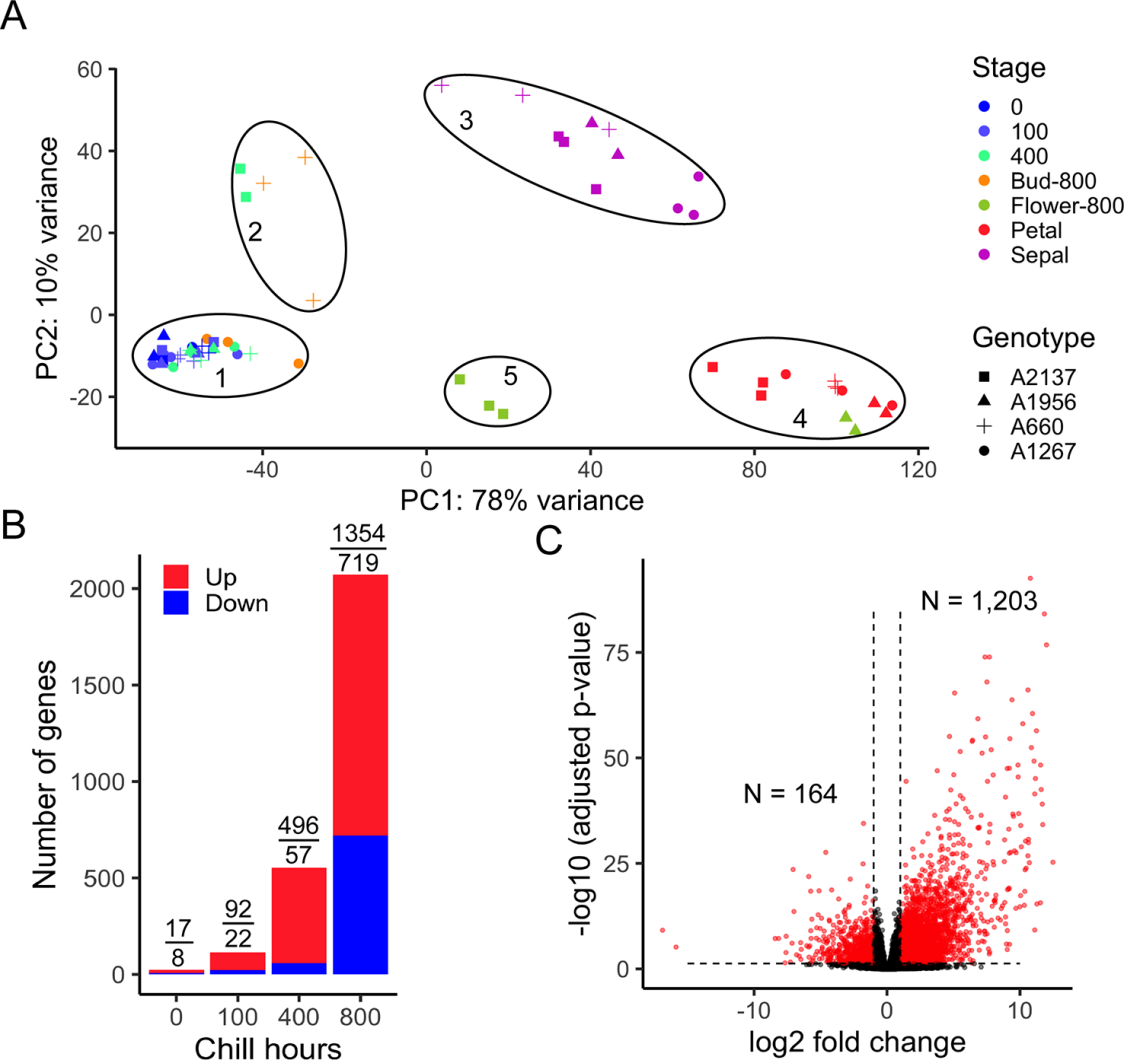
Transcriptome variances distinguish endodormancy and ecodormancy. **(A)** A principal component analysis (PCA) of samples by transcriptome profile in apricot. PC1 and PC2 represent the first two largest sample variances from overall gene expression. The samples were clustered into five groups. 1: endodormancy, 2: ecodormancy, 3: sepal, 4: petal, 5: flower. Biological replicates ranged from 1 to 3 for each genotype and time point (**Supplementary Tables 1** and **2**). **(B)** The number of DEGs between early blooming and late blooming genotypes at 0, 100, 400, and 800 chill hours, with red and blue bars representing the number of upregulated and downregulated genes, respectively. Numbers of DEGs upregulated and downregulated are displayed above the bars. **(C)** Volcano plot of DEGs (red) and non-DEGs (black) (|log2 fold change| > 1, adjusted p-value < 0.05) with 1,203 genes upregulated and 164 genes downregulated at ecodormancy compared to endodormancy.

To evaluate the overall transcriptomes of four apricot genotypes, the sample distances were represented using a principal component analysis (PCA). The first two PCs represented the two largest variances in the gene expression profiles, which explain 78% and 10% of the variance for the first and second PCs, respectively. The 60 transcriptome profiles were clearly separated into five groups (**Figure 2A**) correlated with developmental stages. As CR has not been measured for the four apricot genotypes, the endodormant and ecodormant developmental stages were unknown at the time of collection. However, the PCA plot reveals one main cluster of most dormant bud samples (cluster 1) and a smaller set of dormant bud samples in a separate cluster (cluster 2). Cluster 2 is formed of samples of the earliest blooming genotype (A2137) at 400 hours and one of the later blooming genotypes (A660) at 800 hours. Both the timing and comparison to bloom date data suggest this cluster represents ecodormancy, i.e., as these two genotypes have different bloom dates, the early blooming genotype moved into ecodormancy earlier than the late blooming genotype. Likely, the ecodormant stage was not captured in the other two genotypes. Although it is difficult to distinguish endodormancy and ecodormancy physically from the buds, the gene expression profiles indicated that the trees were progressing towards the same developmental stages but at different rates. From here on the five PCA clusters will be referred to by these development time points: 1- endodormancy, 2- ecodormancy, 3- sepal visible, 4- petal visible, and 5- flower.

To understand the phenotypic effect on dormancy transition between early and late blooming phenotypes, we compared the gene expression of early blooming trees and late blooming trees at each of three time points. After experiencing 0, 100, and 400 chill hours, we found 25, 114, and 553 genes, respectively, were differentially expressed between the two phenotypes (**Figure 2B** and **Supplementary Tables 3–5**), suggesting relatively small differences in the transcriptome profiles at the earliest sampling times. However, at 800 chill hours, early blooming genotypes had passed the bud break stage, while the late blooming genotypes were still in dormancy. At this point, the expression of 2,073 (1,354 upregulated and 719 downregulated) genes significantly changed (**Figure 2B**). However, since different genotypes progressed developmentally at different rates, comparing genotypes at the same time point does not yield information on the developmental stages of dormancy. To investigate further, we compared the expression of genes between endodormancy and ecodormancy stages based on the PCA clusters (regardless of the date of sampling) by contrasting the 28 samples in endodormancy and five samples in ecodormancy. In total, 1,367 out of 26,872 genes were significantly differentially expressed with the cutoff for false discovery rate < 0.05 and an absolute fold change > 2 (|log***_2_***FC| > 1). This analysis yielded 1,203 genes that were upregulated and 164 genes that were downregulated at the ecodormancy stage (**Figure 2C**).

To identify the functions and biological pathways that the DEGs are involved in, GO enrichment analysis was applied. The 1,367 genes differentially expressed between endodormancy and ecodormancy were analyzed by AgriGO v2.0 (Tian et al., 2017), yielding significant GO terms (FDR < 0.05) involved in pathways such as oxidation reduction (GO:0055114, FDR = 0.00075), carbohydrate metabolic process (GO:0005975, FDR = 0.0057), and hydrolase activity (GO:0016787, FDR = 0.014) (**Table 3**). The GO enrichment analysis of 640 DEGs between early and late blooming genotypes at the 0, 100, and 400 chill hours indicated enrichment of GO terms related to cell wall metabolism (GO:0044036, FDR= 0.00089) and chitin metabolic process (GO:0006030, FDR = 0.00089) (**Table 4**). Our results indicate both oxidative stress and cell wall modification are important during the period of CR acquisition during endodormancy. Changes in cell wall structure and components are critical for the processes of growth and stress resistance (Zwiazek, 1991), and previous studies have shown the increase of cell wall formation and reactive oxygen species (ROS) signaling (Jian et al., 1997; Considine and Foyer, 2014) during bud dormancy. Chitinases were reported to be associated with protection against freezing and promote cell wall biosynthesis during dormancy in white spruce (González et al., 2015).

**Table 3:** Significantly enriched (FDR > 0.05) GO terms of DEGs comparing endodormancy and ecodormancy.

GO term	Category	Functional description	Number in DEGs	Number in genome	FDR
GO:0055114	P	Oxidation reduction	107	1195	0.00075
GO:0005975	P	Carbohydrate metabolic process	58	591	0.0057
GO:0004180	F	Carboxypeptidase activity	13	44	0.00012
GO:0004185	F	Serine-type carboxypeptidase activity	13	42	0.00012
GO:0070008	F	Serine-type exopeptidase activity	13	48	0.00018
GO:0016491	F	Oxidoreductase activity	118	1344	0.00018
GO:0008236	F	Serine-type peptidase activity	24	161	0.0007
GO:0017171	F	Serine hydrolase activity	24	161	0.0007
GO:0004553	F	Hydrolase activity, hydrolyzing O-glycosyl compounds	43	377	0.0007
GO:0008238	F	Exopeptidase activity	13	59	0.0011
GO:0016798	F	Hydrolase activity, acting on glycosyl bonds	43	396	0.0013
GO:0005506	F	Iron ion binding	44	423	0.0027
GO:0020037	F	Heme binding	40	385	0.0051
GO:0046906	F	Tetrapyrrole binding	40	386	0.0051
GO:0009055	F	Electron carrier activity	18	129	0.011
GO:0016787	F	Hydrolase activity	144	1945	0.014
GO:0016651	F	Oxidoreductase activity, acting on NADH or NADPH	8	34	0.016
GO:0016705	F	Oxidoreductase activity, acting on paired donors, with incorporation or reduction of molecular oxygen	33	323	0.018
GO:0016209	F	Antioxidant activity	15	106	0.023
GO:0070011	F	Peptidase activity, acting on L-amino acid peptides	43	467	0.024
GO:0003824	F	Catalytic activity	412	6346	0.04
GO:0008233	F	Peptidase activity	43	485	0.045

P. biological processes, F. molecular functions.

**Table 4:** Significantly enriched GO terms of DEGs comparing early blooming and late blooming genotypes in endodormancy time points (0, 100 and 400 chill hours).

GO term	Category	Functional description	Number in DEGs	Number in genome	FDR
GO:0044036	P	Cell wall macromolecule metabolic process	6	20	0.00018
GO:0016998	P	Cell wall macromolecule catabolic process	6	19	0.00018
GO:0006030	P	Chitin metabolic process	5	13	0.00018
GO:0006032	P	Chitin catabolic process	5	13	0.00018
GO:0006026	P	Aminoglycan catabolic process	5	13	0.00018
GO:0006022	P	Aminoglycan metabolic process	5	17	0.00066
GO:0000272	P	Polysaccharide catabolic process	5	22	0.0022
GO:0071554	P	Cell wall organization or biogenesis	10	104	0.0032
GO:0005976	P	Polysaccharide metabolic process	8	91	0.02
GO:0005975	P	Carbohydrate metabolic process	27	591	0.024
GO:0004568	F	Chitinase activity	5	13	0.00082
GO:0004553	F	Hydrolase activity, hydrolyzing O-glycosyl compounds	23	377	0.0033
GO:0016798	F	Hydrolase activity, acting on glycosyl bonds	24	396	0.0033
GO:0016787	F	Hydrolase activity	75	1945	0.011
GO:0008236	F	Serine-type peptidase activity	12	161	0.011
GO:0017171	F	Serine hydrolase activity	12	161	0.011
GO:0003824	F	Catalytic activity	207	6346	0.014
GO:0016684	F	Oxidoreductase activity, acting on peroxide as acceptor	8	95	0.023
GO:0004601	F	Peroxidase activity	8	95	0.023
GO:0070001	F	Aspartic-type peptidase activity	7	84	0.027
GO:0016747	F	Transferase activity, transferring acyl groups other than amino-acyl groups	12	198	0.027
GO:0016209	F	Antioxidant activity	8	106	0.027
GO:0004190	F	Aspartic-type endopeptidase activity	7	84	0.027
GO:0004180	F	Carboxypeptidase activity	5	44	0.027
GO:0004185	F	Serine-type carboxypeptidase activity	5	42	0.027
GO:0004175	F	Endopeptidase activity	14	258	0.03
GO:0070008	F	Serine-type exopeptidase activity	5	48	0.031
GO:0016746	F	Transferase activity, transferring acyl groups	13	237	0.032
GO:0004252	F	Serine-type endopeptidase activity	7	97	0.045

P. biological processes, F. molecular functions.

### Co-Expression Analysis Identified Endodormancy and Ecodormancy Related Genes

**Figure 3:**
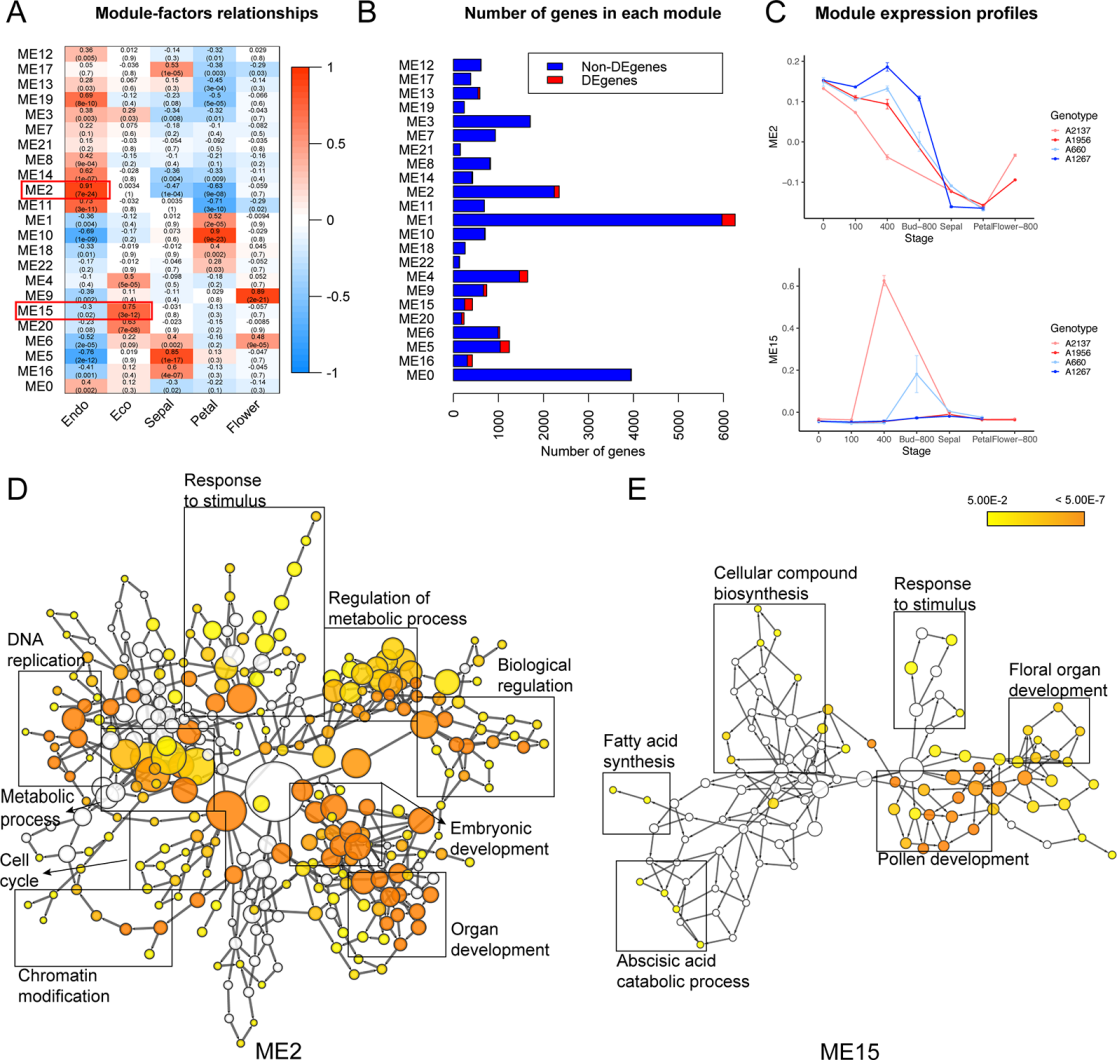
Co-expression modules detected genes induced at endodormancy and ecodormancy. **(A)** The module-factor relationships between modules and developmental stages. **(B)** The number of differentially expressed genes (DEGs) and non-DEGs in each module. **(C)** The expression profiles of ME2 (top) and ME15 (bottom) eigengenes in four apricot genotypes (mean ± SE). The eigengene represents all genes in the module as a single normalized expression pattern. **(D)** The enriched GO network for ME2 genes, and **(E)** the enriched network for ME15 genes. Nodes represent individual GO terms, and node color indicates the *p*-value. General GO categories for the nodes are labeled here with boxes. The individually labeled node networks are available in **Supplementary Figures 4** and **5**. Detailed descriptions of nodes and their *p*-values are in **Supplementary Tables 7** and **9**.

Co-expression clustering was performed to provide better insight into coordinated gene expression modules that contribute to the endodormancy to ecodormancy transition. 25,960 genes detected by RNA-seq were clustered into 23 co-expression modules based on the TOMsimilarity algorithm in the WGCNA R package (Langfelder and Horvath, 2008). Module size ranged from 126 genes (ME22) to 5,967 genes (ME1) (**Figure 3B**). Module-factor relationship analysis correlated the expression level of the modules to the five developmental stages. Higher correlation p-values of a module at a particular stage indicate the genes in that module have higher expression at that stage. According to the module-factor relationship, we identified two modules, module 2 (ME2) and module 15 (ME15) most correlated to dormancy stages (**Figure 3A**). Module 2 is the most positively related to endodormancy and contains 2,350 genes that are expressed highly at the endodormancy stage (**Figures 3A, C** and **Supplementary Table 6**). GO network by BiNGO (Maere et al., 2005) associates the genes in module 2 with biological regulation, stress response, reproduction including embryonic development and cell cycle, metabolic process, and chromatin modification (**Figure 3D** and **Supplementary Table 7**). Module 15 is the most related to ecodormancy (**Figures 3A, C**) and the GO network of the 422 genes in the module indicates that they are associated with hormone biosynthesis including ABA catabolism and secondary metabolism such as terpenoid and isoprenoid catabolic process, as well as flower development such as pollen exine formation, sporopollenin biosynthesis, and anatomical structure morphogenesis (**Figure 3E** and **Supplementary Tables 8** and **9**). The co-expression network patterns indicated that the genes related to pollen and flower development are activated at ecodormancy, suggesting an increase in development of reproductive tissues in the bud at ecodormancy after the CRs are fulfilled.

### Validation of RNAseq by qPCR

To validate our transcriptomic analysis, we performed a reverse transcription qPCR (RT-qPCR) experiment using floral bud RNAs from three early blooming apricot genotypes: A2137, A1956, and A2312. We aimed to validate the RNASeq using the same RNA samples for two of the genotypes (A2137 and A1956), and a previously unexamined early blooming genotype (A2312). A2312 had not previously been analyzed by RNASeq, however, bud samples were collected at the same time points and from the same orchard as the other genotypes. As samples at the 0 chill hour and 100 chill hours were very similar in expression pattern in the previous RNASeq analysis, we chose samples from 100, 400, and 800 chill hours as well as sepal stage for the RT-qPCR validation (**Supplementary Table 2**). The five DEGs with the highest fold change between endodormancy and ecodormancy stage were selected. Prupe.7G084900 is a fatty acyl-CoA reductase, which is associated with male sterility and is involved in oxidoreductase activity and pollen exine formation (Chen et al., 2011). Prupe.2G122600 encodes a member of the chalcone and stilbene synthase family that most closely matches the *Arabidopsis thaliana* gene less adhesive pollen 6 (*LAP6*, AT1G02050), involved in tapetosome development and pollen viability [The Arabidopsis Information Resource (TAIR); Dobritsa et al., 2010; Wang et al., 2018]. Prupe.5G014900 is a glutathione S-transferase, many of which provide cellular detoxification and have been profiled in stress response in many plant species (Seppänen et al., 2000; Anderson and Davis, 2004; Jain et al., 2010). Prupe.1G104900 is a late embryogenesis abundant protein (LEA), a family known to be involved in cold and drought stress response (Pedrosa et al., 2015) and previously profiled in dormant apricot (Yamane et al., 2006) and oak buds (Ueno et al., 2013). The final RT-qPCR gene was *DAM6* (ppa010714m) discussed above.

**Figure 4:**
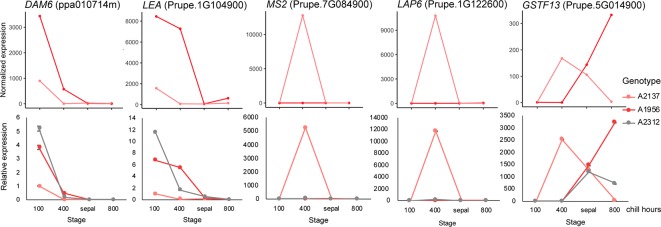
Validation of differentially expressed genes (DEGs) with RT-qPCR. The expression pattern of DEGs in the RNA-seq (top) matched the qPCR (bottom) results. Pink: A2137 (Bakour); red: A1956 (Palsteyn); grey: A2312 (Flamingold). *DAM6*, DORMANCY-ASSOCIATED MADS-box gene 6; *LEA*, Late embryogenesis abundant protein gene; *MS2*, Jojoba acyl CoA reductase-related male sterility gene; *LAP6*, Chalcone and stilbene synthase; *GSTF13*, Glutathione S-transferase family gene. One RNA sample from each genotype at each time point was assayed with three technical replicates.

The RT-qPCR analysis of the five genes all matched the RNASeq results in the two previously sequenced genotypes. The expression pattern for A2312, the newly examined genotype, was similar to the other two early blooming genotypes, confirming the replicability of these results (**Figure 4**). For all three genotypes, the *DAM6* gene was reduced in expression as chill hours accumulated until the release of dormancy at the sepal stage. The expression level of *DAM6* in the genotype A2137, the earliest blooming tree, was already lower at 100 chill hours than that in the other two genotypes. Two of the genes, jojoba acyl CoA reductase-related male sterility protein (Prupe.7G084900) and chalcone and stilbene synthase (Prupe.2G122600), were only induced at the ecodormancy stage in a single genotype, A2137.

### Expression Patterns in Peach Buds Were Similar to Apricot During Dormancy Transition

To confirm the transferability of these results to peach, a similar transcriptome profiling experiment was performed using a set of F_2_ peach trees with different CRs. Four genotypes were selected with low CR and high CR: two genotypes with 300 hours CR (A209 and A340; low CR) and two genotypes with 850 hours and 1,100 hours of CR (A318 and A323, respectively; high CR) (Fan et al., 2010; Zhebentyayeva et al., 2014). Floral buds from the low CR genotypes were collected at 0, 100, 600 chill hours, and pre-bloom, while the high CR genotypes were collected with an additional time point, 1,000 chill hours. Based on imaging of dissected buds, (**Supplementary Figure 6**), the carpels from low CR buds were more mature than those from high CR buds at 600 chill hours while the high CR buds developed similar carpal sizes at 1,000 chill hours (**Supplementary Figure 6**). This result suggests tissue differentiation and development proceeds at different rates based on CR with significant floral development activated at ecodormancy. This agrees with previous reports of cell division and differentiation in anthers during winter dormancy, with microsporogenesis beginning after reaching CR in apricot (Julian et al., 2011).

**Figure 5:**
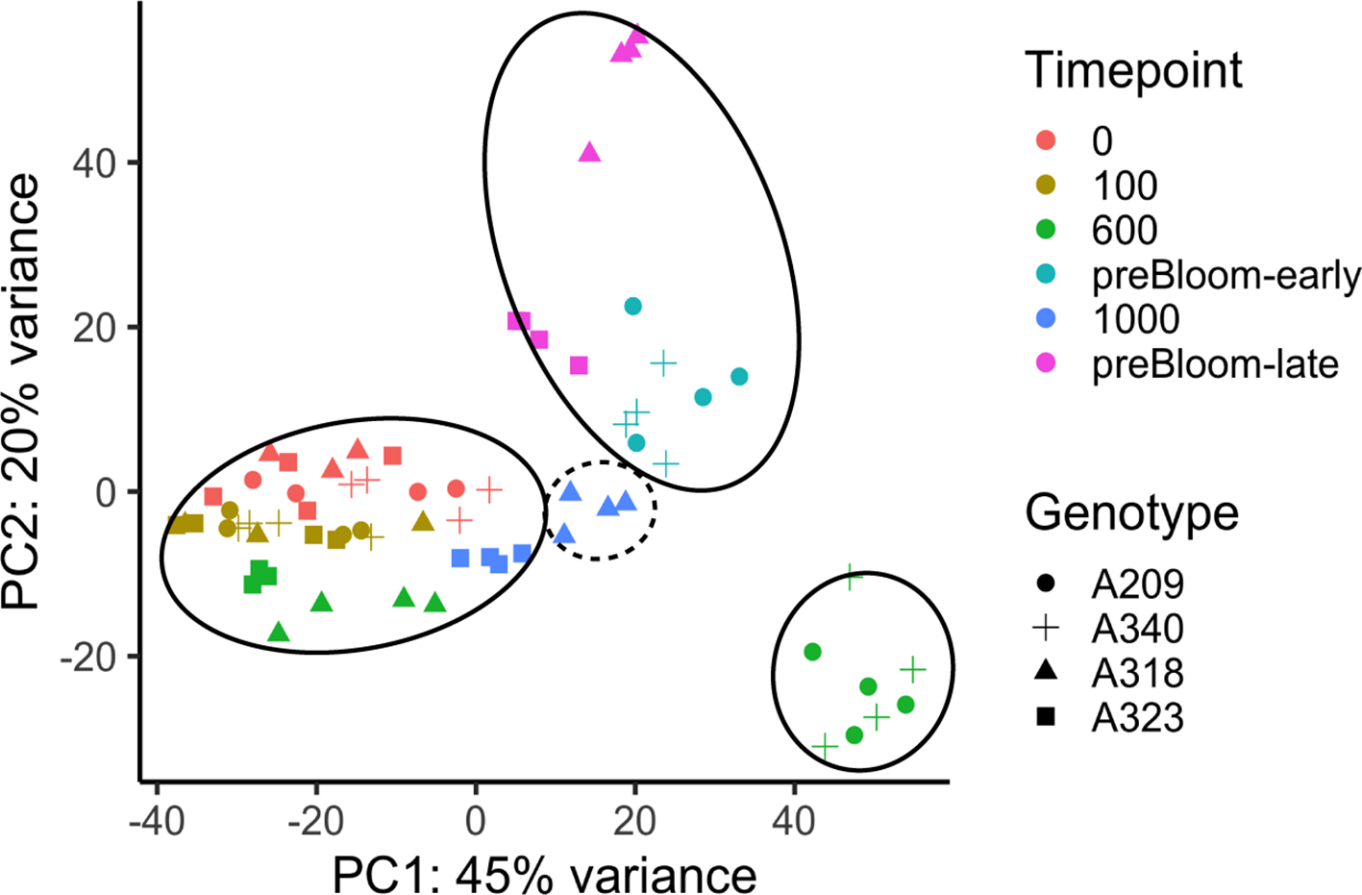
Transcriptome variances of peach floral buds in dormancy during chill accumulation. PCA analysis of peach bud transcriptome profiles. PC1 and PC2 represented the largest two components of variation identified among samples. Solid circles indicate samples at endodormancy (bottom left), ecodormancy (bottom right) and pre-bloom (top) stages. The dashed circle indicates the four A318 samples taken 1,000 chill hours. Despite this genotype having a previously measured CR of 850 hours, these samples cluster more closely with endodormant buds. All genotypes and time points had three or four biological replicates (**Supplementary Tables 1** and **2**).

To examine the changes in transcriptome profiles at sample time points in these peach floral buds, RNASeq analysis was conducted with 69 peach libraries using a similar experimental approach to that of the apricot. Unlike the apricot data, significant ribosomal RNA (rRNA) contamination was identified in more than 50% of the libraries (**Supplementary Figure 7**). To inspect whether the rRNA contamination significantly affected the transcriptome profiles, a PERMANOVA was conducted with transcriptome distance matrices. The result indicated that the percentage of rRNA in the library is a significant factor in the transcriptome variation, but it does not show a significant interaction with the genotype or time point factors (**Supplementary Table 10**). The rRNA content was likely caused by the failure of rRNA depletion during library preparation before sequencing and, based on the statistical results, can be adequately factored out of the experimental analysis. This is further supported by PCA plotting, showing the gene expression profiles clustering into developmental stages regardless of rRNA content (**Figure 5**). In a strikingly similar pattern to the apricot data, the clustering reveals that the low CR buds were in an ecodormant state at 600 chill hours while the high CR buds were still at endodormancy. One genotype may indicate this pattern does not perfectly correspond to endo- and ecodormancy. Buds at 1,000 chill hours from genotype A318 with 850 CR (i.e. putatively ecodormant) were clustered more closely to other endodormant buds (**Figure 5**). Whether the buds were actually endodormant or ecodormant can only be predicted from chill hours and was not directly assayed. However, the overall transcriptome profile variations in peach and apricot both indicate two distinct transcriptional programs that largely correspond to endodormancy and ecodormancy. To ensure that the differential gene expression analysis of peach samples clearly represents these two stages, the differential expression analysis was performed on samples from the 600 hour time point, in which both the PCA-based clustering and predicted chill hours for each cultivar both agree on the endodormant and ecodormant stage of the samples.

The expression profiles of the *DAM* genes were comparable in peach and apricot. Similar to apricot *DAM5* and *DAM6*, peach *DAM5* and *DAM6* were expressed at a high level at the beginning of endodormancy then downregulated after 100 chill hours. However, a different expression profile for *DAM3* was found between the species. In peach, *DAM3* shared the same expression pattern with *DAM4*, generally upregulated in endodormancy, then downregulated at ecodormancy (**Figure 1** and **Supplementary Figure 8**). This is different from *DAM3* in apricot, which is consistently downregulated from the beginning of dormancy to the end.

Transcriptome profiles for low chill and high chill genotypes were compared to identify the DEGs at each time point. As expected, at 0 and 100 chill hours, when low chill and high chill genotypes were both in endodormancy, few gene expression differences were found: 49 DEGs at 0 chill hour and 77 DEGs at 100 chill hours (**Supplementary Figure 9A**). At the time point of 600 chill hours, when the two low chill genotypes moved into ecodormancy and the high chill genotypes were still in endodormancy, 2,102 genes were differentially expressed, with 1,603 upregulated and 499 downregulated (**Supplementary Figures 9A, B**). Four of the six *DAM* genes (*DAM3*, *DAM4*, *DAM5,* and *DAM6*) were included in the DEGs (**Supplementary Table 11**). The DEGs were enriched in the pathways of oxidation reduction process (GO:0055114, FDR = 1.01e-08), carbohydrate metabolic process (GO: 0005975, FDR = 1.7e-07), and cell wall metabolic process (GO: 0044036, FDR = 4.89e-04), which were also enriched in the apricot DEGs (**Supplementary Figure 9C**). These results confirm that the genes involved in stress response, sugar metabolism, and cell wall assembly contribute to the endodormancy to ecodormancy transition, and that this pattern of expression is replicable across at least two *Prunus* species.

### Comparison of Peach and Apricot Transcriptome Profiles

**Figure 6:**
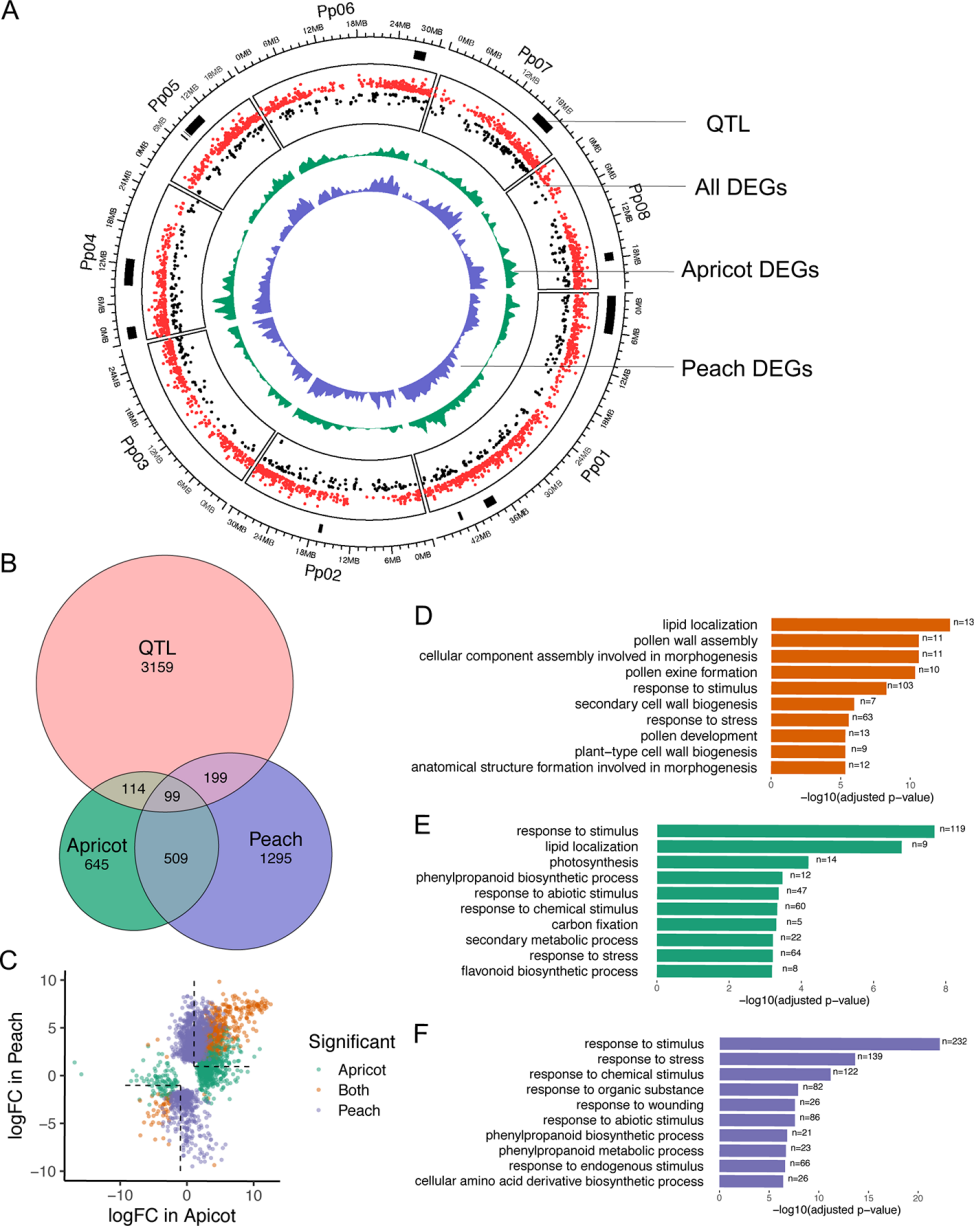
Comparison of differentially expressed genes (DEGs) between endodormancy and ecodormancy in apricot and peach. **(A)** The distribution of DEGs and quantitative trait loci (QTLs) on the reference genome. Red and black dots represent up and down fold changes, respectively. **(B)** Venn diagram of apricot and peach DEGs overlapping with peach CR QTLs. **(C)** A comparison between DEGs in apricot and peach based on the log2-fold change (logFC). Colors indicate genes that were statistically significant in apricot only (green), peach only (purple), or both (orange). **(D–F)** Top 10 GO terms enriched in DEGs found in both species **(D)**, peach only **(E)**, and apricot only **(F)**.

To compare peach and apricot, we considered only the DEGs found to differentiate endodormancy and ecodormancy stages. The DEGs in both apricot and peach were distributed across all eight chromosomes (**Figure 6A**). Of the 1,367 DEGs identified in apricot and the 2,102 DEGs in peach, 608 genes were statistically significant in both species (**Figure 6B**). When comparing with the fold change of DEGs in peach and apricot, over 99% of the DEGs were either upregulated or downregulated consistently in both species, with only six genes expressed inconsistently (**Figure 6C**, **Supplementary Table 11**). Prupe.8G238000, Prupe.6G047900, and Prupe.5G057900 were downregulated at ecodormancy in apricot but were upregulated in peach. Prupe.3G259300, Prupe.4G069300, and Prupe.3G127600 were upregulated in apricot but downregulated in peach. These very consistent patterns between peach and apricot gene expression suggest there is a highly conserved gene regulatory system governing dormancy stages and transitions across *Prunus* spp. as well as a few potentially important variations.

CR and bud break QTLs have previously been identified in both apricot and peach linkage maps (Olukolu et al., 2009; Fan et al., 2010; Zhebentyayeva et al., 2014). Using these genetic mapped positions, we sought to filter our DEGs for possible candidate genes in these QTL regions. For apricot, there are 1,059 markers [43 simple sequence repeats (SSR) and 1,016 amplified fragment length polymorphism (AFLP)] located on the Perfection and A.1740 linkage map (Olukolu et al., 2009). The detected CR QTLs regions were delimited by AFLP markers, which were unable to be located in the peach reference genome due to a lack of marker-associated sequence data. For peach, 15 QTLs associated with CR from a cross of peach cultivars “Fla.92-2C” and “Contender” (Fan et al., 2010; Zhebentyayeva et al., 2014) encompass a total of 3,571 genes. These genes were extracted to compare with apricot and peach DEGs. Of 1,367 apricot DEGs, 213 were detected in the QTLs while 298 out of 2,102 peach DEGs overlapped with these QTLs (**Figure 6A**). A final set of 99 genes was identified as being located within the QTL regions and statistically significant in dormancy-associated expression changes in both apricot and peach (**Figure 6A**). *DAM4* (ppa011123m), *DAM5* (ppa010822m), and *DAM6* (ppa010714m) were part of this final set and were the only genes located on QTL qCR1a-2009.

In order to broadly understand the functions of DEGs in peach and apricot individually as well as their shared functions, a GO enrichment analysis was conducted for three sets of genes: the shared DEGs (608), the DEGs unique to apricot (759), and the DEGs unique to peach (1,494). When examining GO term enrichment of the DEGs shared in both peach and apricot, significantly enriched terms include lipid localization (GO:0010876, FDR=1.5e-13), response to stimulus (GO:0050895, FDR=5.3e-9), pollen wall assembly (GO:0010208, FDR=2.6e-11), and cellular component assembly involved in morphogenesis processes (GO:0010927, FDR=2.6e-11) (**Figure 6D**). The 2,253 DEGs identified in either peach or apricot but not both, were enriched in the same biological pathways as the DEGs that were only significant in one species. Despite the two lists not sharing any genes, when examining the unique peach DEGs and the unique apricot DEGs, four of the top 10 enriched GO terms based on the lowest FDR values were shared (**Figures 6E, F**). This shared set of GO terms suggests that peach and apricot share many of the same functions and pathways during dormancy stages and transitions, even if different individual genes are being identified as differentially expressed. For example, 232 DEGs involved in stimulus response were in peach but not in apricot, while 119 stimulus response genes were differentially expressed in apricot but not in peach. A similar situation was found for phenylpropanoid biosynthetic pathway, response to abiotic stimulus, and response to chemical stimulus, i.e. they are significant in both species but mostly consisted of different underlying genes from peach and apricot. In contrast, pollen and floral development genes tended to be found as DEGs in both species, and thus GO terms relevant to those processes appear on the shared list.

There are pathways that were enriched only for one species but not for the other. For example, GO terms for photosynthesis and carbon fixation were significant in apricot. Fourteen and five DEGs, respectively, were found to be associated with those GO terms (**Figure 6E**), and all are found in the chloroplast genome. A different set of five DEGs involved in photosynthesis was found in the peach transcripts, but was not significantly differentially expressed between stages. While chloroplasts occur in green floral tissues such as sepals, they have also been found to be photosynthetically active in other floral organs such as petals and corollas in other plants (Weiss et al., 1989; Pyke and Page, 1998). Chloroplasts have also been proposed as essential for redox processes and developmental signaling during flower development (Muñoz and Munné-Bosch, 2018).

### Comparison of Co-Expression Networks Between Apricot and Peach

Similar to apricot co-expression networks, peach bud transcriptome profiles distinguished co-expressed genes that were upregulated at endodormancy or ecodormancy, respectively. ME6, the cluster of genes with the highest expression level at endodormancy was identified as positively related to endodormancy stage (correlation = 0.63). (**Supplementary Figure 10A**). The co-expression networks by Cytoscape indicated that the ME6 genes were enriched in phenylpropanoid biosynthetic process (GO:0009699, FDR=0.024), regionalization (GO:0003002, FDR=0.0319), and positive regulation of flavonoid biosynthetic process (GO:0009963, FDR=0.0319) pathways (**Supplementary Figure 10C** and **Supplementary Table 12**). ME4 and ME10, were both identified as positively related with the ecodormancy stage (ME4 correlation = 0.94, ME10 correlation = 0.75) (**Supplementary Figures 10A**, **B**). ME4 genes were enriched in organic acid catabolic process (GO:0016054, FDR=7.59e-7), oxoacid metabolic process (GO:0043436, FDR=9.68e-7), and response to stimulus (GO:0050896, FDR=9.68e-7). ME10 genes were enriched in pollen wall assembly (GO:0010208, FDR=1.19e-11), cellular component assembly involved in morphogenesis (GO:0010927, FDR=1.19e-11), and pollen exine formation (GO:0010584, FDR=1.86e-11) pathways (**Supplementary Figures 10C**, **D**). Both significant and insignificant GO descriptions are provided in the **Supplementary Tables 12–14**.

**Figure 7:**
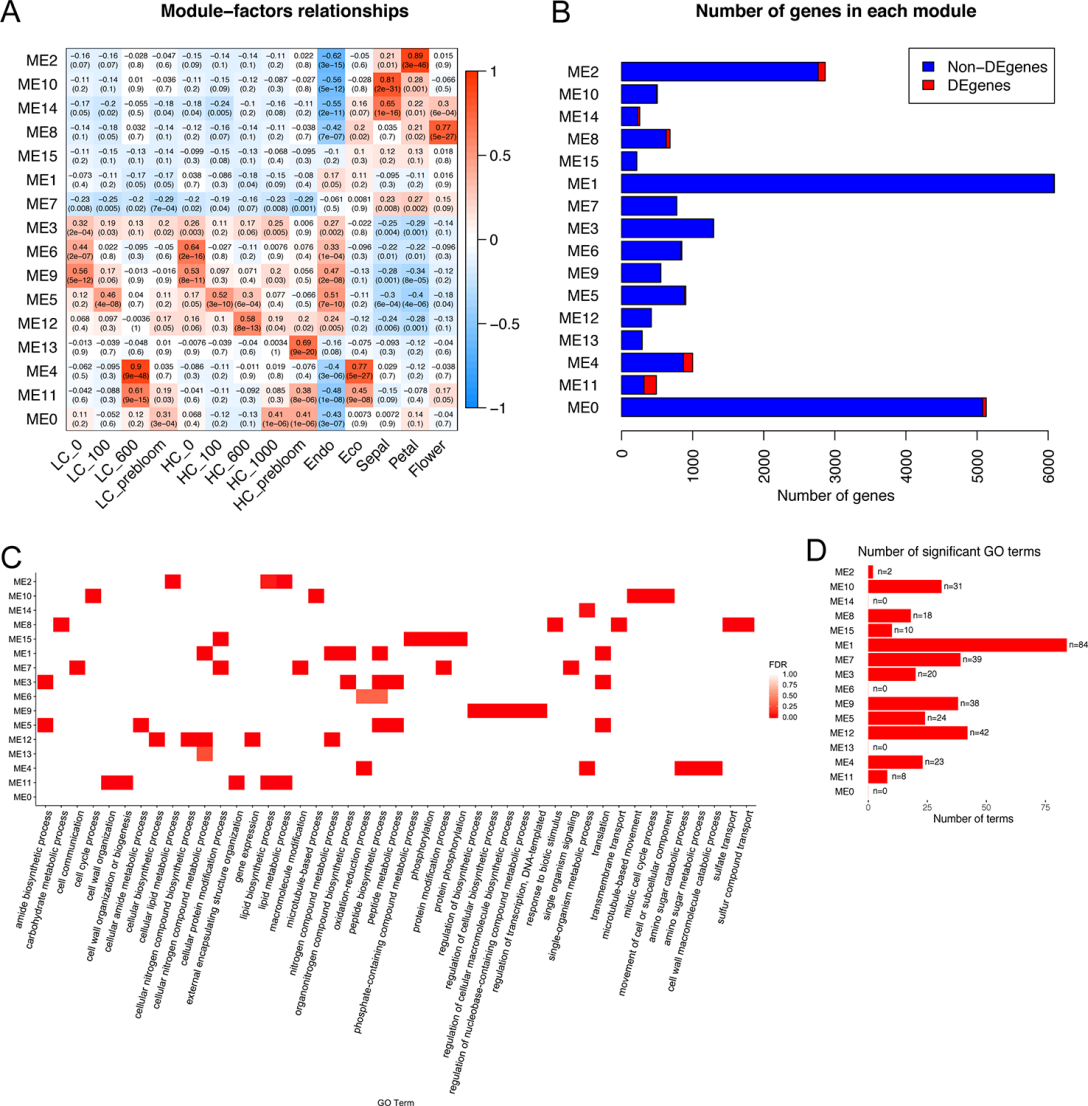
Co-expression modules identified genes induced commonly at endodormancy and ecodormancy in peach and apricot. **(A)** The module-factors relationships with the correlation of module eigengenes (MEs) with time points and developmental stages. LC, low chill; HC, high chill. **(B)** The number of genes in each module, including differentially expressed genes (DEGs) (in red) shared in both species and non-DEGs (blue). **(C)** A heatmap with the top 5 enriched gene ontology (GO) terms for each module. **(D)** The number of significant GO terms shared in both apricot and peach in each module.

Co-expression networks developed independently from the peach and apricot data identified genes involved in many shared pathways, such as pollen wall assembly and stress response. To further investigate the common expression patterns in both apricot and peach, a co-expression analysis integrating the peach and apricot datasets was performed. Twenty-two thousand three hundred seventeen genes expressed in more than half of the samples in the dataset were clustered into 16 modules spanning from 215 genes (ME15) to 6,089 genes (ME1). DEGs were distributed unevenly across the 16 modules, with the largest number as 174 DEGs in ME11, and no DEGs in ME7 or ME15 (**Figure 7B**). To understand the major biological processes that genes are involved in for each module, genes were then enriched by AgriGO v2.0 using the *Prunus persica* background. Three hundred thirty-nine significant GO terms from the biological process category were enriched across the 16 modules. ME1 had the most with 84 enriched GO functions, while ME0, ME6, ME13, and ME14 had no significant GO terms (**Figure 7D**). Most of the modules do not share the same GO terms, suggesting multiple genes in important dormancy-related biological functions are co-expressed in similar patterns across our experimental time points. For example, cell wall macromolecule catabolic process (GO:0016998) was only enriched in ME4, while cell cycle process (GO:0022402) and mitotic cell cycle process (GO:1903047) were only enriched in ME10.

**Figure 8:**
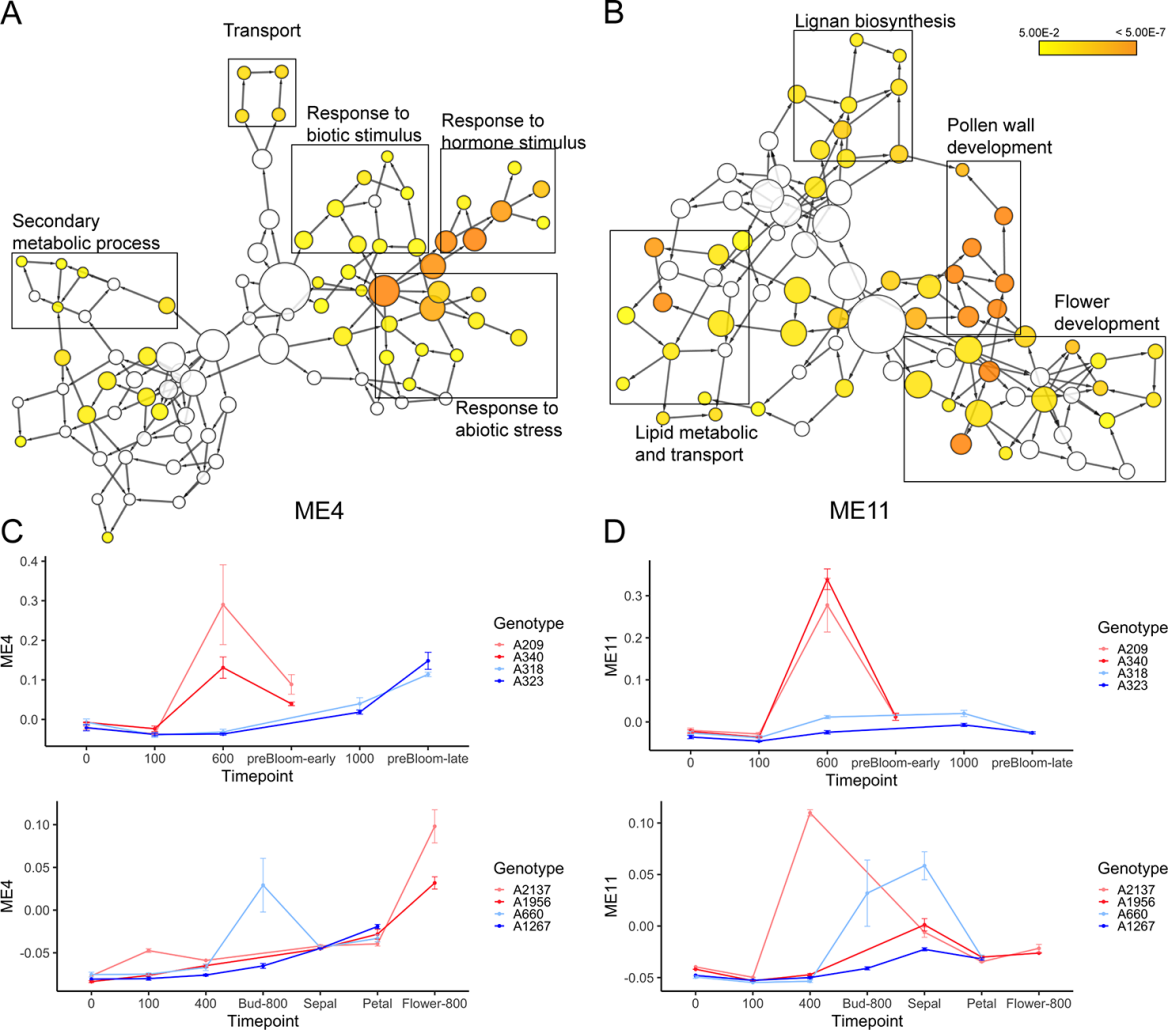
The enriched gene ontology (GO) networks for ME4 **(A)** and ME11 **(B)**. **(C)** The expression profiles of the ME4 eigengene (mean ± SE) in peach (top) and apricot (bottom). **(D)** The expression profiles of the ME11 eigengene (mean ± SE) in peach (top) and apricot (bottom).

Two modules, ME4 and ME11, are highly correlated to ecodormancy in peach (correlation = 0.9) and apricot (correlation = 0.77) in the co-expression analysis (**Figure 7A**). A total of 35.5% of the genes in the ME11 were DEGs shared between apricot and peach, and of 1,000 genes in ME4, 129 were shared DEGs (**Figure 7B**). These two modules contained half of the shared DEGs (303 out of 608), suggesting that genes in these two modules are essential to defining transcriptional patterns in ecodormancy. The expression profile of the ME4 eigengene showed an upregulation at ecodormancy in peach low chill genotypes but up-regulation in only one apricot genotype, A660 (**Figure 8C**). The expression of ME11 eigengene was upregulated at ecodormancy in both peach and apricot genotypes, however, the eigengene in apricot A660 continued increasing at the sepal stage (**Figure 8D**). The GO enrichment network of ME4 indicates the genes are involved in response to abiotic and biotic stimuli and response to hormones (ethylene, abscisic acid, gibberellin, and jasmonic acid). A group of genes in ME4 were involved in organic acid transport and secondary metabolic processes, specifically sesquiterpenoid metabolism (**Figure 8A** and **Supplementary Table 15**). These pathways indicate the ME4 genes are most likely responding to the abiotic stress imposed during winter (**Supplementary Table 15**). The other module, ME11, is enriched in pollen wall development, flower development, lignan metabolism, and lipid transport (**Figure 8B** and **Supplementary Table 16**). These results suggest that both peach and apricot activate new floral development activities including pollen wall and flower morphogenesis after CRs are fulfilled.

## Discussion

*Prunus* flowers are sensitive to frost damage, and maintenance of winter dormancy for a sufficient time period is essential to prevent frost damage to delicate reproductive tissues, especially during the spring months when temperatures may fluctuate. This is one of the most important adaptation mechanisms that ensure perennial plants survive low winter temperatures and coordinates the timing of reproductive activities (Campoy et al., 2011; Luedeling et al., 2011; Leida, 2012). There have been many efforts to find the possible regulation factors of winter dormancy in perennial trees, including examinations of internal factors such as phase change, circadian clock, and hormones, and external factors including photoperiod, temperature, light radiation, and water (Perry, 1971; Rohde and Bhalerao, 2007; Allona et al., 2008; Cooke et al., 2012; Maurya and Bhalerao, 2017). Our time-series transcriptome profiles in apricot and peach buds allowed us to investigate the gene expression changes as floral bud dormancy progressed towards bloom and to compare complementary data from two closely related species and among genotypes varying in CR/BD.

### Buds Stay Active During Winter Dormancy

Meristems are the functional units for plant development, including root and shoot apical meristems, as well as cambium and intercalary meristems (Esau 1965). Dormancy of buds at the cellular level is defined as eco-developmental quiescence of a shoot meristematic organ, whereby tissues fail to respond to the environment (Considine and Considine, 2016). However, based on our transcriptome profiles and imaging of internal tissues, buds continued development during endodormancy. With the co-expression analysis in this study, results showed that cell cycle genes were upregulated during endodormancy (**Figure 3** and **Supplementary Figure 4**). For example, the co-expression module with the highest correlated score to endodormancy stage in apricot (ME2) included cyclin D genes (Prupe.1G430500, Prupe.6G229700, and Prupe.8G146700) and cell cycle checkpoint control protein (Prupe.6G321300), indicating cells were actively dividing during endodormancy. Cell division can be induced by cytokinins through increasing cyclin expression (Dewitte et al., 2007). A cytokinin mediated gene, cytokinin response factor 2 (*CRF2*, Prupe.5G114100) in apricot buds was co-expressed with cyclin D genes, indicating potentially high levels of cytokinins during endodormancy. Treatment of a synthetic cytokinin has been shown to terminate potato tuber dormancy through downregulation of the AGAMOUS-like MADS-box transcription factor, a homolog to the peach *DAM* genes (Campbell et al., 2014). CRF mutants reduced pistil and ovulve growth (Cucinotta et al., 2016) and *CRF2* is transcriptionally induced by cold stress in *Arabidopsis* (Jeon et al., 2016), implying cytokinin signaling may play a critical role in modulating cell division in cold. High expression levels of cell cycle genes and cytokinin response genes during endodormancy suggest that cells in the dormant floral buds were actively dividing and/or differentiating. A previous study of anatomical development of floral structures in peach buds reported slow but continuous differentiation throughout the winter, especially of androecium and gynoecium tissues (Reinoso et al., 2002). Our bud images show increasing definition of floral structures (**Supplementary Figure 6**).

### Epigenetic Modification Plays an Important Role in Dormancy

One set of major dormancy regulators known in fruit trees are *DAM*s, with previous studies reporting that increased trimethylation of H3K27 and decreased H3K4 trimethylation represses the expression of *DAM*s during dormancy (Leida, 2012; Leida et al., 2012b; Saito et al., 2015a). In our study, all six copies of *DAM* genes decreased at the transition from endodormancy to ecodormancy, and were maintained at a low level after ecodormancy (**Figure 1** and **Supplementary Figure 8**). The co-expression networks identified a group of genes co-expressed with *DAM1* (ppa018667m) and *DAM3* (ppa010758m), and that were involved in chromatin modification and organization (**Figure 3D** and **Supplementary Figure 4**), including Prupe.2G042400 and Prupe.1G177800 related to histone H3K4 methylation, Prupe.4G091400, and Prupe.6G153800 and Prupe.1G533300 related to production of small RNA involved in gene silencing. However, none of these genes were differentially expressed between endodormancy and ecodormancy stages. Studies reported H3K4 trimethylation was enriched during bud break in pear (Anh Tuan et al., 2016) and a transcription factor EARLY BUD-BREAK 1 (*EBB1*) associated with H3K4 positively regulated bud break in poplar (Yordanov et al., 2014), confirming that histone methylation is involved in winter dormancy and bud break of woody trees. Small RNA profiles in Japanese pear have also been shown to be associated with bud dormancy transition (Bai et al., 2016), however, whether the epigenetic effects are causative or consequential remains unclear.

### Genes Responsive to Cold Stress Play a Key Role in Maintaining Endodormancy

One major function of endodormancy is resistance to cold temperatures and the protection of new tissues developing inside of buds. Cold-response genes were identified as DEGs during the endodormancy to ecodormancy transition in apricot and peach. For example, Prupe.2G294400 is a catabolite activator protein (CAP160), which has previously been associated with cold stress response (Kaye et al., 1998). The expression of Prupe.2G294400 initially increased as chilling accumulated and then declined once ecodormancy was reached (**Supplementary Figure 11**), and this expression pattern was consistently observed in all four genotypes of both species. This mirrors experiments in other plants, for example, expression of *CAP160* transcripts in spinach increased when plants were exposed to low temperatures, and a transgenic tobacco overexpressing *CAP160* displayed a higher tolerance to freezing stress (Kaye et al., 1998). Similar to the CAP160, one *LEA* gene (Prupe.1G104900) was significantly downregulated during ecodormancy but increased during chill hour accumulation in endodormancy (**Figure 4** and **Supplementary Figure 11**). The LEA protein family has been shown to respond to abiotic stress such as cold, drought and salt (Hara et al., 1999; Chandra Babu et al., 2004; Checker et al., 2012; Duan and Cai, 2012; Pedrosa et al., 2015). The *LEA* expression pattern in our experiments is consistent with previously observed *LEA* gene expression, which was induced by short photoperiod and downregulated as dormancy progressed (Jiménez et al., 2010a; Leida et al., 2012a).

*CBF*s are known to respond to cold and drought in plants and are responsible for 10%–20% of transcriptional changes induced by cold (Stockinger et al., 1997; Vogel et al., 2005; Medina et al., 2011; Shi et al., 2018). Two *CBF*s were significantly downregulated during the ecodormancy stage in peach but not in apricot (**Supplementary Table 11**). Overexpression of *CBF* in peach delayed bud break and induced *LEA* genes and other cold-induced genes (Wisniewski et al., 2015; Artlip et al., 2019). CBFs bind upstream of *DAM* genes and induce their expression at early time points (Niu et al., 2016; Zhao et al., 2018). While we did see upregulation of *DAM* genes 4 and 5 in apricot and *DAM* genes 3, 4, 5, and 6 in peach at early time points especially in late blooming genotypes, all *DAM* genes were relatively highly expressed at the beginning of the experiment, prior to chilling that would induce CBFs. These patterns match previous *Prunus DAM* gene expression profiles (Li et al., 2009; Yamane et al., 2011) and may indicate regulatory mechanisms other than CBF that operate prior to chill. Cold-responsive (*COR*) genes are also responsive to CBF signals and are induced by low temperatures, in order to increase plant cold tolerance, leading to cold acclimation (Nylander et al., 2001; Chinnusamy et al., 2007; Chinnusamy et al., 2010). Peach Prupe.7G161100, an *AtCOR* ortholog, was differentially expressed between dormancy phases in peach but not apricot. It also shared similar expression patterns with the *LEA* gene (Prupe.1G104900) (**Supplementary Figure 11**). The induction of cold responsive genes including *COR*, *LEA*, and *CAP160* during endodormancy suggests that these genes may contribute to cold tolerance of flower buds during endodormancy. The up-regulation of cold tolerance genes lasted longer in the high chill genotypes than in the low chill genotypes in peach, suggesting cold tolerance is consistently maintained throughout endodormancy. In a process analogous to vernalization, it has been proposed that these genes may also contribute to sensing the amount of chill accumulation during endodormancy (Shi et al., 2018). However, after trees fulfilled their CR and moved into ecodormancy, the genes were decreased to a low level of expression, despite the continuing vulnerability to frost or freeze damage during ecodormancy. Therefore, these particular cold responsive genes may only respond to cold and induce tree cold tolerance during endodormancy. Other mechanisms may be involved in combating cold stress during ecodormancy. As these trees are grown outside their climatically adapted ranges, this could also be due to a misalignment of the phenology of the trees with the locale and would be interesting to investigate further in native populations.

### ROS Responsive Genes Are Differentially Expressed Between Endodormancy and Ecodormancy

Oxidative stress response has been found during dormancy and is hypothesized to be not only a stress response but also an important signaling mechanism (Considine and Foyer, 2014). Our results showed that 63 DEGs found in both *Prunus* species were enriched in response to stress, which included oxidation stress (**Figure 6**). Co-expression networks of apricot and peach also indicated ME4, which was induced during ecodormancy, was enriched in genes involved in the oxidation reduction process (**Figure 7C**). The production of ROS, especially hydrogen peroxide (H_2_O_2_) during endodormancy release was identified as the major signal that triggered antioxidation pathways, such as an increase of peroxidase and superoxide dismutase (SOD) (Leida et al., 2010; Tan et al., 2010; Viti et al., 2010; Prassinos et al., 2011; Vergara et al., 2012; Bai et al., 2013). In our analyses, six oxidoreductase genes (Prupe.2G288400, Prupe.3G168200, Prupe.5G089100, Purpe.6G262200, Prupe.8G156700, and Prupe.8G195100) and five peroxidase family proteins (Prupe.1G081600, Prupe.6G191900, Prupe.6G192000, Prupe.6G239400, and Prupe.7G035800) were significantly upregulated during ecodormancy in both species; two of them (Prupe.1G081600 and Prupe.5G089100) are within the peach CR QTL regions (qCR1d-2008 and qCR5-2009, respectively). Previous studies have shown that exogenous application of H_2_O_2_ can induce endodormancy break in Japanese pear (Kuroda et al., 2005); an increase of H_2_O_2_ was also observed in sweet cherry and peach after applying hydrogen cyanamide, a chemical that induces dormancy-break (Pérez et al., 2008; Ionescu et al., 2017). Our results using field-grown apricot and peach trees found an upregulation of antioxidant defense genes, consistent with the findings from hydrogen cyanamide induced dormancy break (Tang et al., 2019). This supports the hypothesis that ROS induces endodormancy release, which consequently induces bud break. The upregulation of antioxidants and other defense genes likely function as protection from the increase of ROS during dormancy phase transition, which was observed in both apricot and peach buds.

### Flower Development-Related Genes Have Specific Patterns of Expression Corresponding to Endodormancy and Ecodormancy Stages

DEGs and co-expression clusters included a number of genes involved in flower development that are upregulated during ecodormancy. Jojoba acyl CoA reductase-related male sterility proteins (Prupe.7G084900), chalcone and stilbene synthase (Prupe.2G122600 and Prupe.8G159600), Pollen Ole e 1 allergen and extensin family proteins (Prupe.4G061100 and Prupe.3G204300), and gamete expressed protein 1 (Prupe.1G133700) were identified as DEGs in both species, and were clustered into ME11 where gene expression was induced at ecodormancy (**Figure 7D**, **Supplementary Tables 11** and **16**). Previous studies reported stamen and microspore development after dormancy break in apricot flower buds, and sweet cherry anthers showed developmental activation after CR fulfillment (Julian et al., 2011; Fadón et al., 2019). Pollen development-related genes have also been shown to be upregulated after dormancy break in peach cultivars (Ríos et al., 2013).

Besides the upregulated DEGs at ecodormancy, genes involved in flower development were also found to be downregulated during ecodormancy. Prupe.1G388300, an ortholog of VERDANDI (*VDD*), which is a transcription factor that regulates female gametophyte differentiation in *Arabidopsis* (Matias-Hernandez et al., 2010), was differentially expressed in both apricot and peach. *VDD* was upregulated in endodormancy during chill accumulation and then downregulated at the ecodormancy stage (**Supplementary Figure 12**). In *Arabidopsis*, this gene is induced by ovule identity MADS-box transcription factor complexes consisting of SEEDSTICK (STK), SEPALLATA3 (SEP3), and SHATTERPROOF during ovule development (Favaro et al., 2003). However, the expression of *VDD* in peach and apricot was opposite to the expression of *STK*, as *STK* (Prupe.1G549600) was upregulated from dormancy transition to flowering (**Supplementary Table 11** and **Supplementary Figure 12**). This suggests that *VDD* and female gametogenesis may be regulated by other factors rather than STK complexes during dormancy in *Prunus* spp. Another jojoba acyl CoA reductase-related male sterility protein (Prupe.6G126800) involved in flower wax biosynthesis (Busta and Jetter, 2017) and ovate family protein 2 (OFP2, Prupe.6G290900) involved in vascular formation (Schmitz et al., 2015; Wang et al., 2016) were also downregulated during ecodormancy, indicating that some floral development activities may be completed prior to entering ecodormancy. Julian et al. (2011) reported a rapid development of stamen and vascular differentiation upon CR fulfillment in apricot (Julian et al., 2011). These transcriptional patterns, coupled with our images of developing buds and previous work, demonstrate that floral development progresses in stages closely tied to dormancy progression.

### The DEGs Located in QTL Regions May Be Involved in Dormancy Regulation

Of the shared DEGs for apricot and peach, 99 were located within the CR QTL regions, including three of the *DAM* genes (*DAM4*, *DAM5*, and *DAM6*) in the QTL qCR1a-2009. However, these three *DAM* genes were clustered into two different co-expression modules. *DAM4* fell into ME1, which did not show obvious up or down-regulation during CR accumulation in either peach or apricot, although the *DAM4* gene was downregulated during dormancy release (**Figure 1** and **Supplementary Figure 13**). *DAM5* and *DAM6* were clustered into ME5, where genes were expressed highly at 0 chill hour and decreased during dormancy release (**Supplementary Figure 13**).

Other DEGs overlapping with CR QTLs include genes involved in transcriptional regulation, sugar transportation, stress response, and cell wall development. As a response to abiotic stress, the heat shock transcription factor A-2 (*HSFA2*, Prupe.7G206900) found on LG 7 QTL qCR7-2008 was upregulated at ecodormancy. *HSFA2* accumulates under heat stress, similar to other heat shock proteins (Schramm et al., 2006). The targets of HSFA2 include the HSP70 (70-kDa heat shock) protein family (Prupe.7G108000 and Prupe.7G108400), which were also upregulated during ecodormancy in apricot. This agrees with a previous study reporting an induction of HSP70 before floral bud break in Japanese pear (*Pyrus pyrifolia*) (Takemura et al., 2015). However, some other heat shock proteins such as Hsp21, Hsp83, and Hsp40 were downregulated during ecodormancy in both apricot and peach, in contrast to the expression patterns of *HSP70* (**Supplementary Table 11**).

Three orthologs of sugar transporter protein 1 (Prupe.5G083900, Prupe.5G090900, and Prupe.5G091100 in LG5 QTL qCR5-2008 and qCR5-2009), which are regulated by sugar levels in plants, were significantly upregulated during ecodormancy in both apricot and peach, indicating that the sugar concentrations were likely increased during ecodormancy. This is consistent with a study in Japanese apricot (*Prunus mume*), which found that soluble sugars such as glucose and sucrose were at low levels at the beginning of dormancy, and gradually increased until dormancy release (Zhang et al., 2018). Sugar transportation and synthesis are important to budbreak in Japanese pear, Japanese apricot, and cherry (Marafon et al., 2011; Zhuang et al., 2015; Chmielewski et al., 2017; Fadón et al., 2018a). Dormant cherry buds accumulate starch throughout endodormancy and chilling induces the increase of starch and sucrose concentrations in Japanese pear buds (Marafon et al., 2011; Fadón et al., 2018a). Sugar transport may also have an active role in regulating dormancy transitions, as transgenic poplar expressing *Arabidopsis* sucrose phosphate synthase exhibits early bud flush (Park et al., 2009). The activity of α-amylase which hydrolases starch into sucrose is reported to increase after floral and vegetative buds release from endodormancy (Hussain et al., 2015). To resume growth from dormancy, buds not only use local sugars, but also transport carbohydrates from long distances (Tixier et al., 2019). Tixier et al. (2017) propose long-distance sugar transport in walnut sustains the fast growth resumption after dormancy release (Tixier et al., 2017). In this study two beta-galactosidase genes found in the QTLs qCR7-2008 and qCR-2009 (Fan et al., 2010), *BGAL7* (Prupe.7G194500), and *BGAL1* (Prupe.7G210000), were downregulated during ecodormancy in both apricot and peach. BGAL enzymes produce free galactose, which is the most dynamic sugar residue of the cell wall during fruit development in tomato (Smith and Gross, 2000). Down-regulation of *BGAL* during ecodormancy also supports the idea that the bud is increasing cell wall formation activities during ecodormancy. In *Arabidopsis* thaliana, BGAL7 and BGAL8 have been profiled as increasing expression during bud development, particularly in stamens and mature pollen for BGAL7 (Schmid et al., 2005; Berardini et al., 2015).

### Plant Hormones Are Involved in the Endodormancy to Ecodormancy Transition

Co-expression networks identified genes involved in signaling pathways of growth-related hormones during ecodormancy, including ABA responsive genes and GA responsive genes (**Figure 8A** and **Supplementary Tables 11** and **15**). Two GA 2-oxidase genes (Prupe.4G080700 and Prupe.4G204600) were significantly downregulated during ecodormancy in both apricot and peach (**Supplementary Table 11**). GA 2-oxidase is the enzyme controlling the bioactive GA levels during plant development. It deactivates GA_1_ and GA_4_, two major bioactive GAs in plants (Yamaguchi, 2008). Downregulation of GA 2-oxidase genes may increase the bioactive GA levels after the transition from endodormancy to ecodormancy, reactivating plant growth. In *Prunus mume*, GA_3_ concentrations were found to increase during the dormancy phase transition, and the low chill genotype had higher GA_3_ levels than the high chill genotype during ecodormancy (Wen et al., 2016). The GA biosynthesis gene GA 20-oxidase was also expressed at a high level during ecodormancy (Wen et al., 2016). Unlike *Prunus mume*, there were no significant changes in GA 20-oxidase gene in our study in either peach or apricot, suggesting that the increase of GA levels in peach and apricot may be due to the suppression of GA deactivation rather than an increase of GA biosynthesis. In *Populus*, chilling was found to increase GA biosynthesis and signaling genes, leading to a suggested model where GA is required for dormancy release and acts by inducing removal of callose thus reopening pores and plasmodesmata for signaling to resume growth (Rinne et al., 2011).

ABA has an opposite effect as GA on dormancy. Our co-expression networks among peach and apricot identified three genes involved in ABA metabolic process (GO:0009687, FDR=0.0258), and 15 genes responsive to ABA stimulus (GO:0009737, FDR=1.71E-04); all were upregulated during ecodormancy. Among these 18 genes, two Myb-containing domain genes *Myb108* (Prupe.1G111700) and *Myb102* (Prupe.4G192000) and one homeobox 7 (*HB7*, Prupe.3G316600) were differentially expressed in both species. Past studies reported that HB7 is a stress response protein positively regulated by the ABA pathway (Söderman et al., 1996; Georgii et al., 2019). The high expression level of *HB7* transcripts during ecodormancy in apricot and peach suggests high concentrations of ABA, which may be acting to repress bud break during ecodormancy. However, the two Myb proteins are shown to inhibit ABA accumulation and down-regulate ABA signaling (Cui et al., 2013; Piao et al., 2019). These genes may indicate counteracting or balancing influences on the ABA pathway during ecodormancy, to promote dormancy release and bud growth.

The small auxin up RNA (SAUR)-like auxin responsive gene family was also significantly upregulated during ecodormancy in both species. The upregulation of six *SAUR-like* genes (Prupe.2G194600, Prupe. 7G167000, Prupe.8G080300, Prupe.8G081100, Prupe.8G081700, and Prupe.8G157800) indicated that auxin levels may be increasing during ecodormancy compared to during endodormancy, consistent with the results found in grapevine that auxin concentrations rise from ecodormancy until budburst (Aloni et al., 1990). Moreover, the downregulation of auxin efflux carrier genes (Prupe.1G071800 and Prupe.5G004300) and upregulation of auxin influx carrier gene (Prupe.1G503100) indicated that increased auxin transporters contribute to increasing auxin levels. Auxin transport has been proposed as a regulatory gate for dormancy control in apple (Porto et al., 2015). Our analyses identified auxin efflux carrier Prupe.1G071800 as a DEG located on a peach CR QTL qCR1d-2008, making it a strong candidate as a dormancy regulator in peach as well.

### Potential Implications

**Figure 9:**
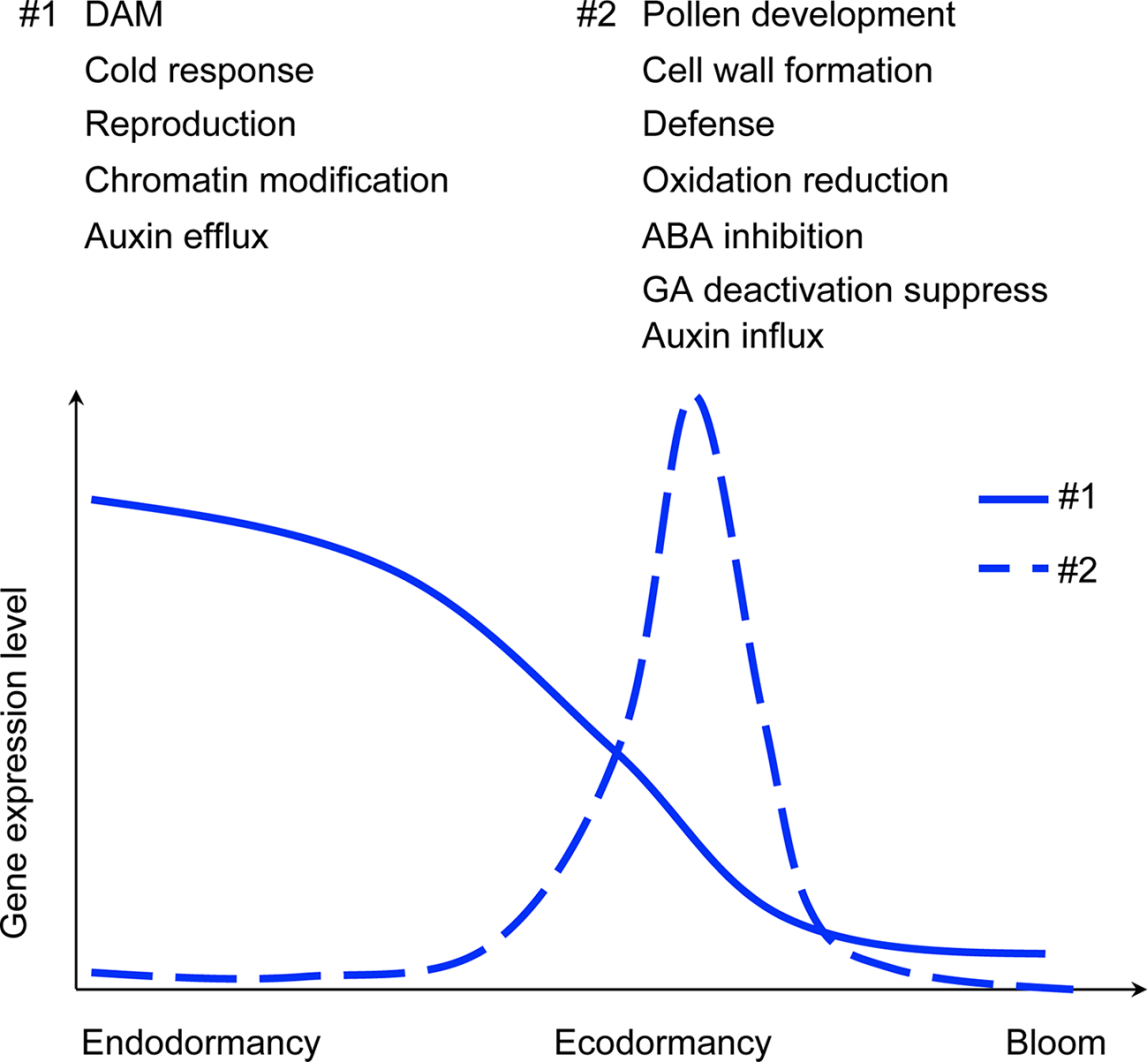
Diagram of two distinctive expression patterns during dormancy phase transition.

The seasonal transition from endodormancy to ecodormancy is a complex and tightly coordinated network of many processes, including environmental sensing, cold acclimation, abiotic stress response, hormone fluctuations, chill hour accumulation, and floral development. Examining the transcriptional patterns at multiple time points during dormancy in two species from two locations each with phenotypically varying genotypes enabled us to elucidate strong, reproducible patterns governing dormancy. Our co-expression networks and DEGs begin to untangle this process at the transcript level into two major gene expression patterns shown in **Figure 9**: 1) gene expression was gradually downregulated as dormancy progresses; 2) low gene expression during endodormancy that then peaks at ecodormancy. Functional annotation and gene ontology enrichment analysis defined the genes in the first pattern as involved in pathways including reproduction and chromatin modification. Genes expressed in the second pattern are involved in pollen development, cell wall formation, defense systems, oxidation-reduction, and hormone metabolic and transport pathways. Genes following the first pattern are likely responsible for chill accumulation and release of endodormancy, while genes following the second pattern may be responsible for preparing the bud for the final steps of floral maturation, such as development of pollen. These patterns form a framework of conserved biological pathways that determine winter dormancy and spring flower timing. This transcriptome knowledge contributes to the overall understanding molecular controls of phenological traits, which is particularly critical for tree crops that suffer major crop losses due to warm winters followed by late frosts in spring when trees have already bloomed and are damaged.

### Future Directions

Our analyses using time-series transcriptomic profiles from dormant buds over winter in peach and apricot identified 99 genes located on the CR QTL region and differentially expressed in both species. These candidate genes need to be examined in mapping populations for sequence or structural variants that might yield phenotypic differences and their transcriptional profiles examined against more dormancy time points. Further, the same material needs to be experimentally verified for the specific endo-vs-ecodormancy transition point. The biological pathways identified by the co-expression networks also point to chromatin remodeling and cell cycle pathways during chilling in the endodormancy stage as likely factors regulating dormancy transition and warrant additional research. The finding of increasing defense response at ecodormancy only in peach also needs further validation in other *Prunus* species.

## Data Availability Statement

All reported raw read data is available at the National Center for Biotechnology Information (NCBI) under the study of project accession PRJNA567655. Raw reads for apricot samples are available from the Sequence Read Archive (SRA) with accession IDs SAMN12791244 to SAMN12791303. Peach samples are available under the accession IDs SAMN12791304 to SAMN12791372. The raw counts of reads per gene derived after read mapping were deposited to NCBI Gene Expression Omnibus (GEO) with accession GSE138792.

## Author Contributions

AA, CD, ZL, MS, TZ, and VD conceived the project. VD, GR, and J-MA collected apricot samples and VD and AA extracted RNA. AC and TZ collected peach samples and CD, ZL, and DB extracted RNA. ZL and DB provided the peach bud imaging. DW performed RT-qPCR. JY and MS performed the data analyses and wrote the manuscript with contributions from all co-authors. All authors approved the manuscript.

## Funding

This project is supported by the Agriculture and Food Research Initiative Competitive Grant No. 2016-67014-24577 from the USDA National Institute of Food and Agriculture (Albert G Abbott, Christopher Dardick, Zongrang Liu, Margaret E Staton) and the French ANR CHEX ABRIWG No. ANR-11-CHEX-0002 (Albert G Abbott, Véronique Decroocq, and Jean-Marc Audergon).

## Conflict of Interest

The authors declare that the research was conducted in the absence of any commercial or financial relationships that could be construed as a potential conflict of interest.
