# Supplementary material for: Distinctive Gene Expression Patterns Define Endodormancy to Ecodormancy Transition in Apricot and Peach
Source: Front Plant Sci. 2020 Feb 28;11:180. doi: 10.3389/fpls.2020.00180 (PMC7059448; doi:10.3389/fpls.2020.00180)
Supplement: Supplementary file 1 [file DataSheet_1.docx]

Supplementary Material

# Supplementary Tables

Table 1. Metadata for apricot and peach RNASeq with NCBI SRA accession

Table 2. Summary of biological replicates for apricot and peach samples

Table 3. Apricot DEGs between early and late blooming genotypes at 0 chill hour

Table 4. Apricot DEGs between early and late blooming genotypes at 100 chill hours

Table 5. Apricot DEGs between early and late blooming genotypes at 400 chill hours

Table 6. Genes in apricot co-expression module ME2

Table 7. GO enriched terms of apricot ME2

Table 8. Genes in apricot co-expression module ME15

Table 9. GO enriched terms of apricot ME15

Table 10. PERMANOVA analysis of factors for peach transcriptome data

Table 11. DEGs in apricot and peach, respectively

Table 12. GO enriched terms of peach ME6

Table 13. GO enriched terms of peach ME4

Table 14. GO enriched terms of peach ME10

Table 15. ME4 GO enriched terms of integrated apricot and peach RNASeq

Table 16. ME11 GO enriched terms of integrated apricot and peach RNAseq

# Supplementary Figures


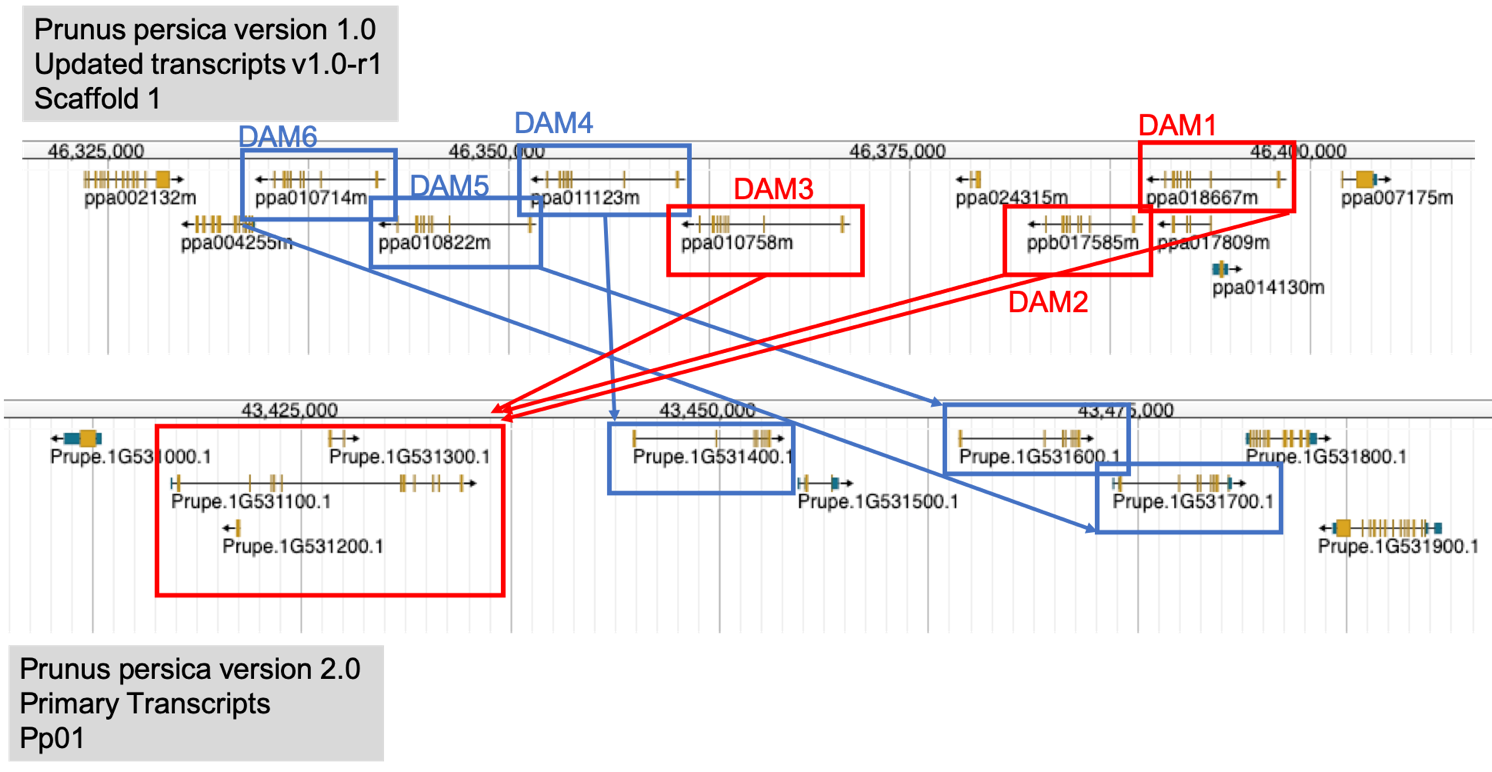


**Supplementary Figure 1.** The misannotation of DAM 1 – 3 in Prunus persica genome version 2.0. The entire region has been reversed in orientation along the chromosome (likely correctly), but the exons from DAM 1-3 are merged into a single large gene with a few exons separated into smaller genes (red lines and boxes).


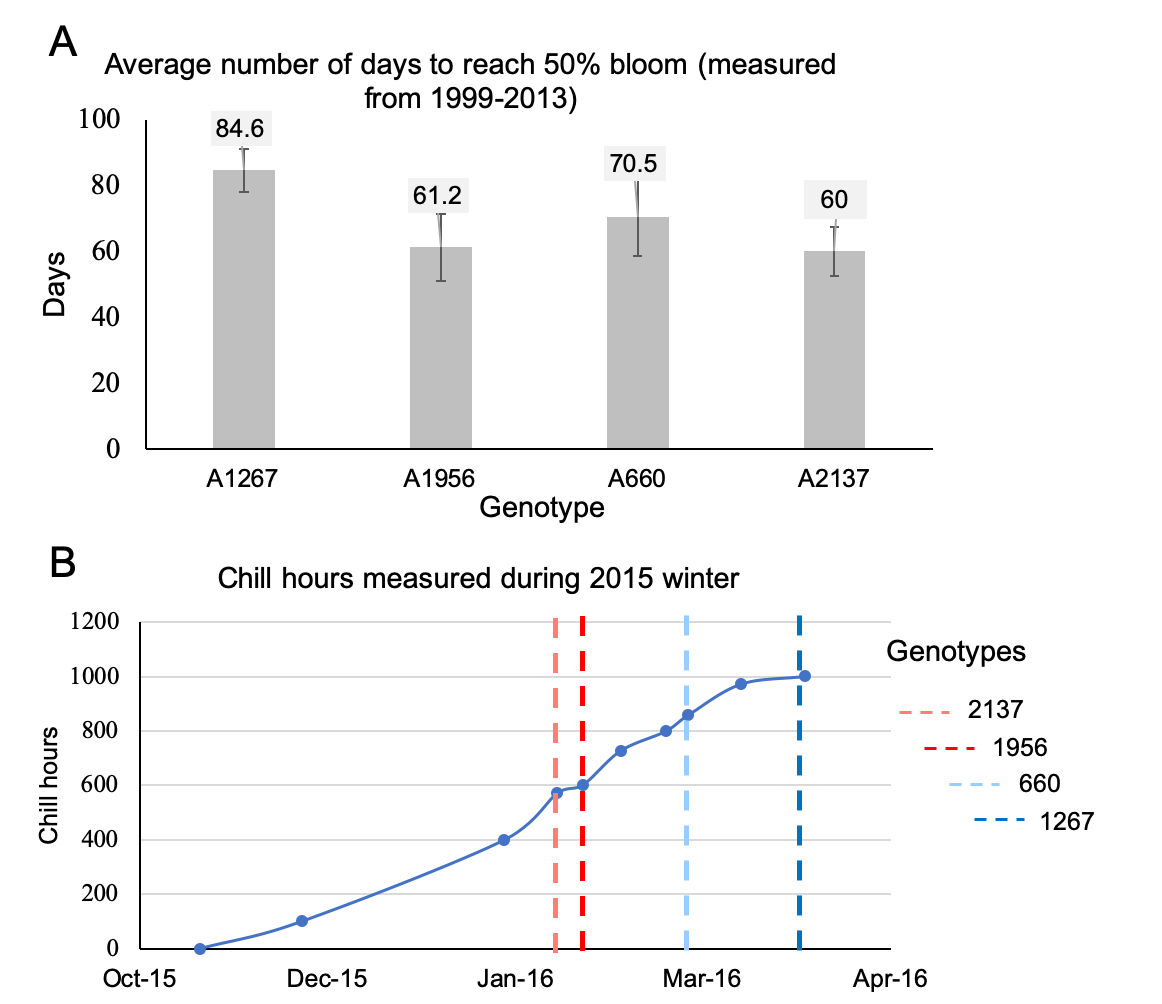


**Supplementary Figure 2.** Phenotypes measured for each Apricot genotype. (A) Days until 50% blooming for four apricot genotypes, averaged from measurements from 1999 to 2013 (Mean±SD). (B) Chill-hour accumulation in the year of sample collection and the sepal visible time for the four genotypes (vertical lines).


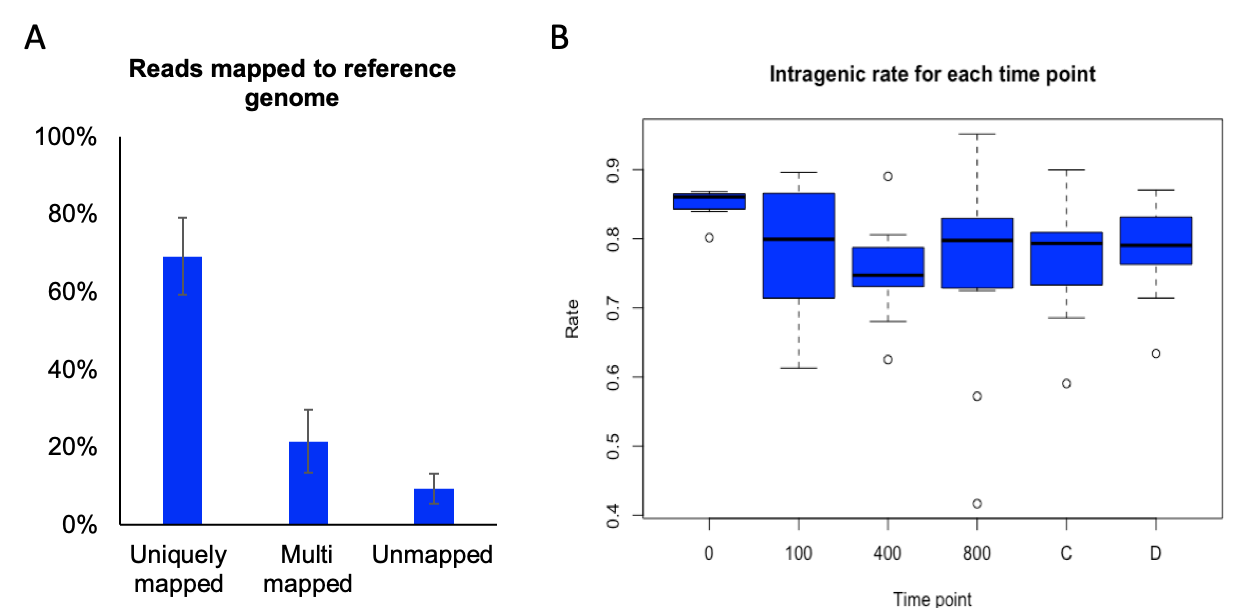


**Supplementary Figure 3**. The mapping rates of apricot samples. (A) Overall mapping rate of apricot RNA-seq samples (Mean+SD). (B) Intragenic rate of apricot RNA-seq reads mapped to peach genome v2.0. (C, sepal visible stage, D, petal visible stage)


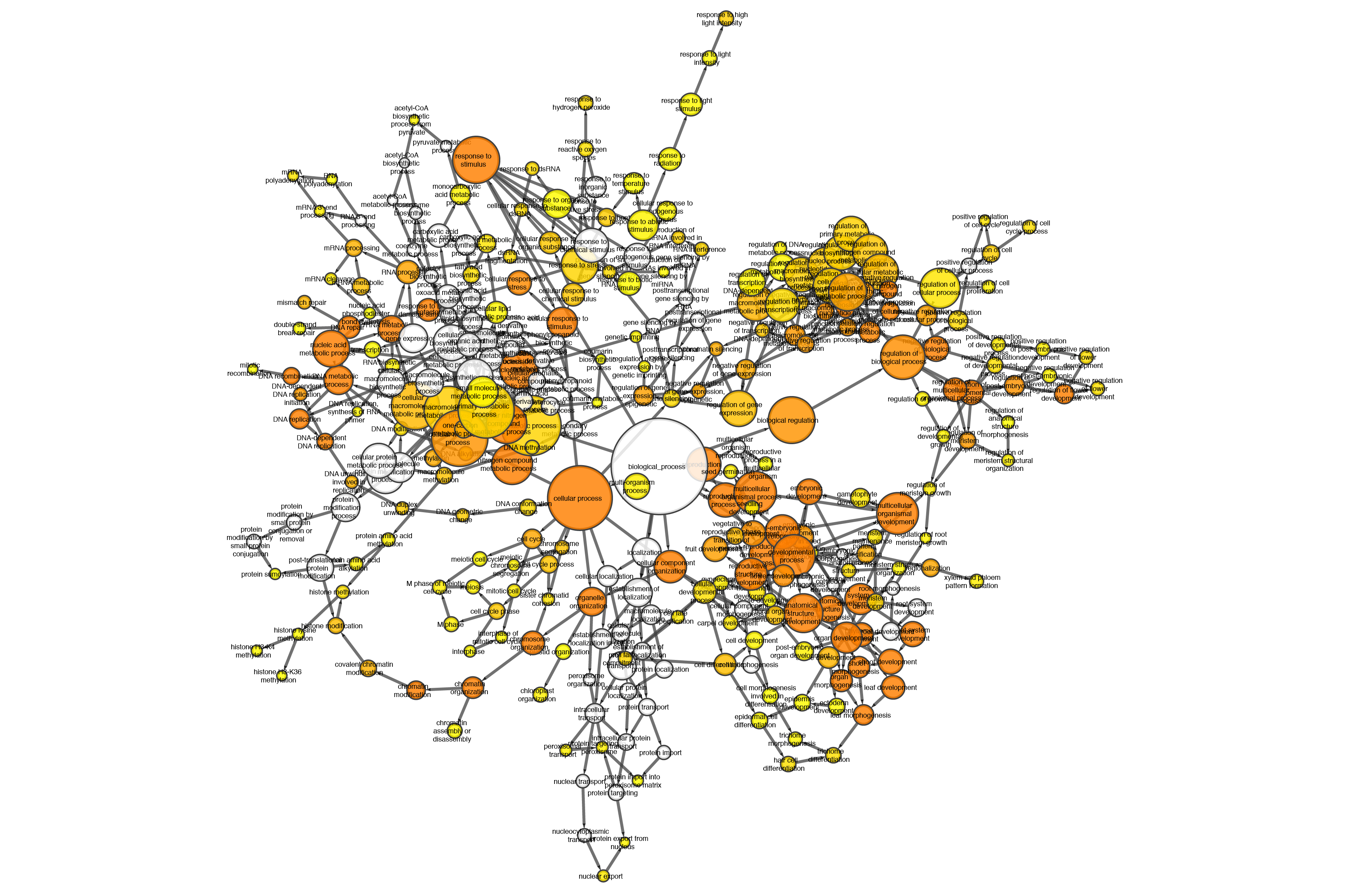


**Supplementary Figure 4**. GO enriched network in apricot Module 2 with labels.


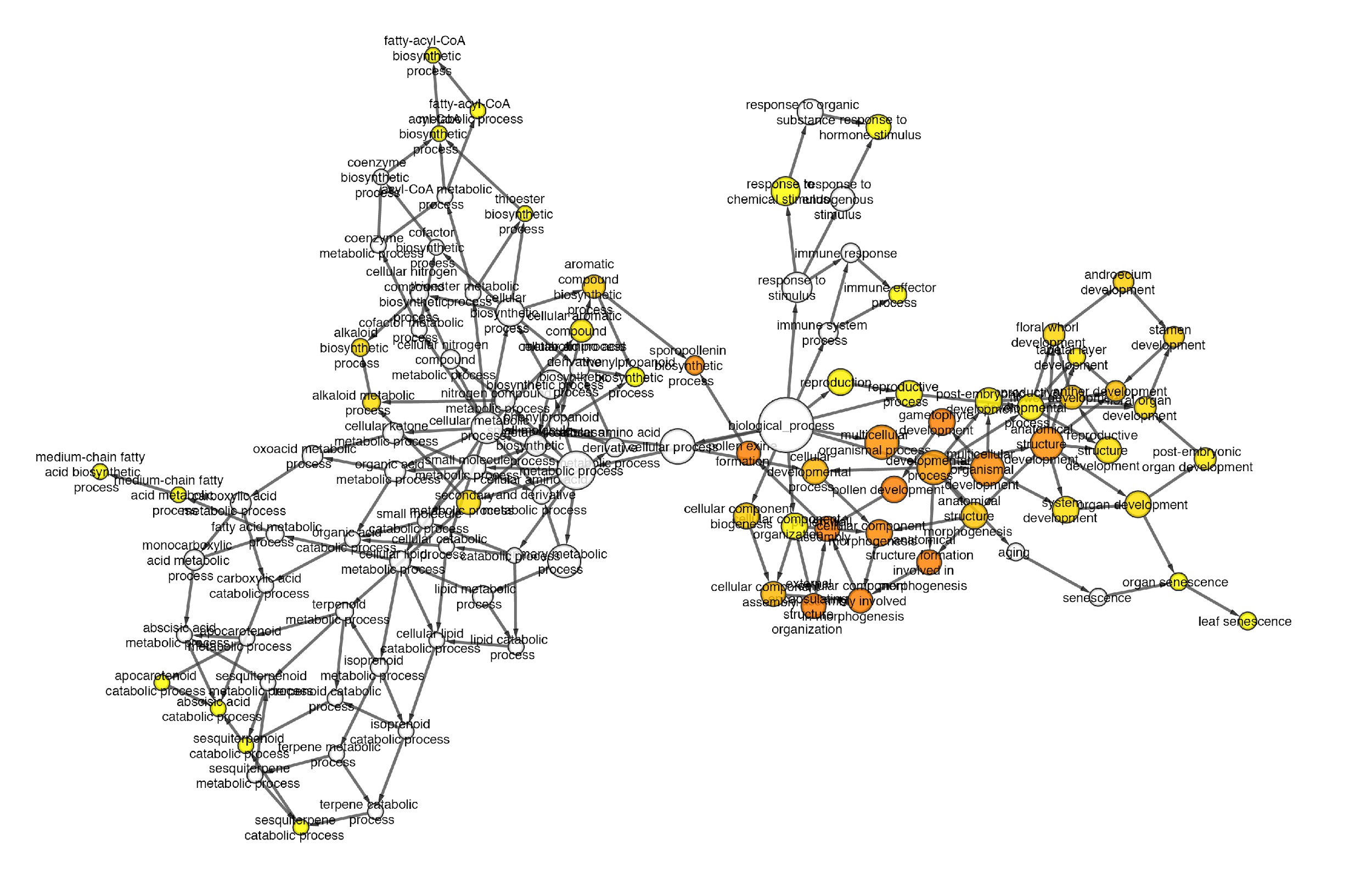


**Supplementary Figure 5**. GO enriched network in apricot Module 15 with labels.


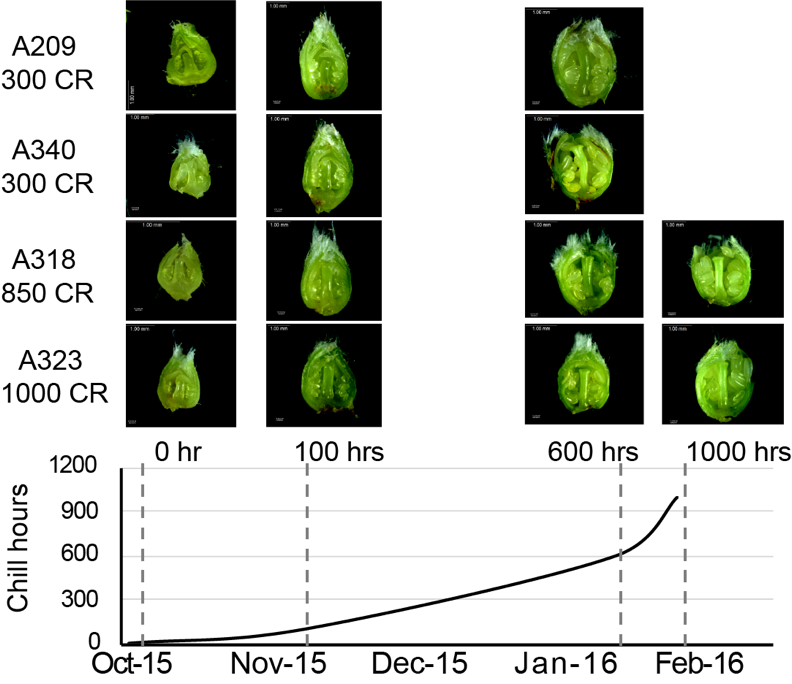


**Supplementary Figure 6**. A representative image of dissected floral buds from each of four peach genotypes at each sampling time point before pre-bloom.


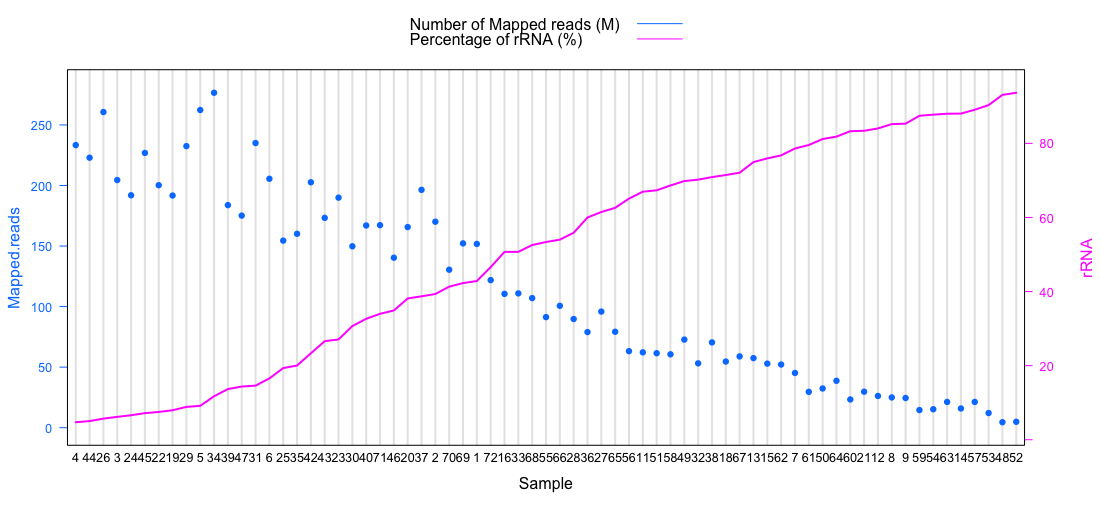


**Supplementary Figure 7**. The effect of rRNA contamination on read alignment. Number of reads aligned to the reference genome (blue) compared with percentage of rRNA (purple) in the libraries.


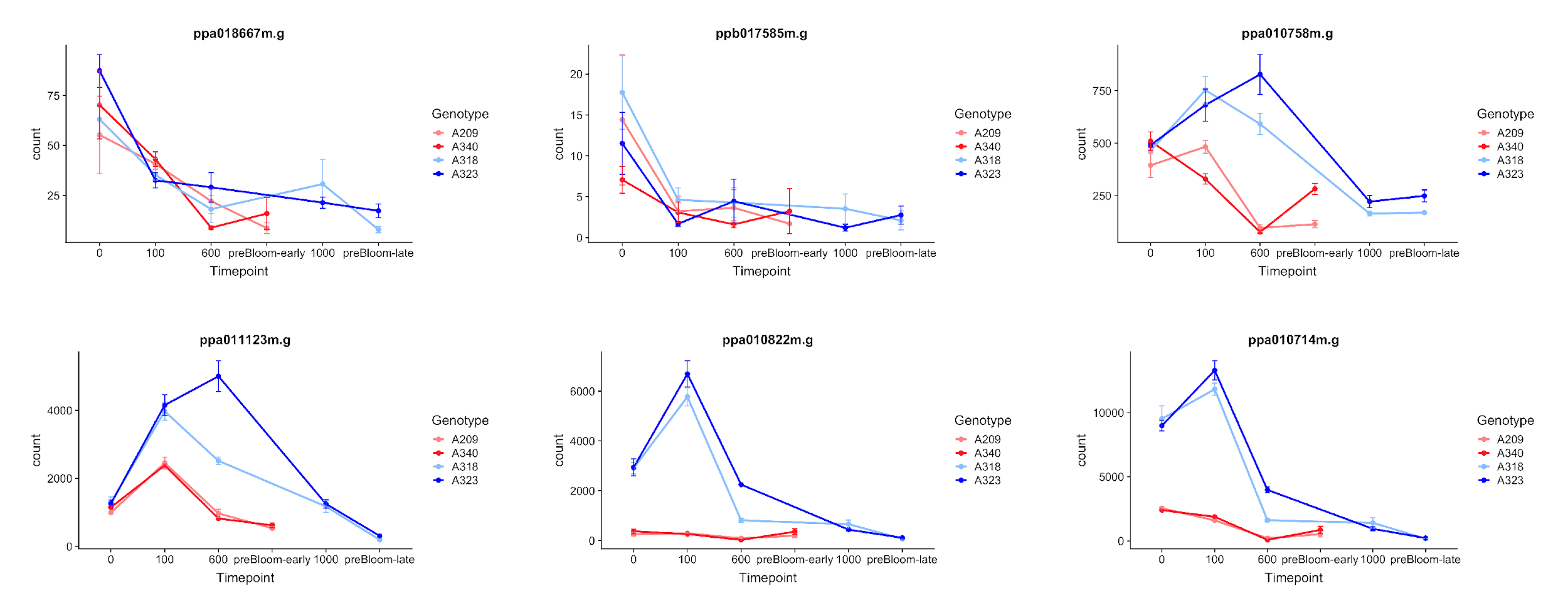


**Supplementary Figure 8**. The expression profiles of six DAM genes in peach during dormancy (Mean+SE). Top, DAM1-DAM3; bottom, DAM4-DAM6.


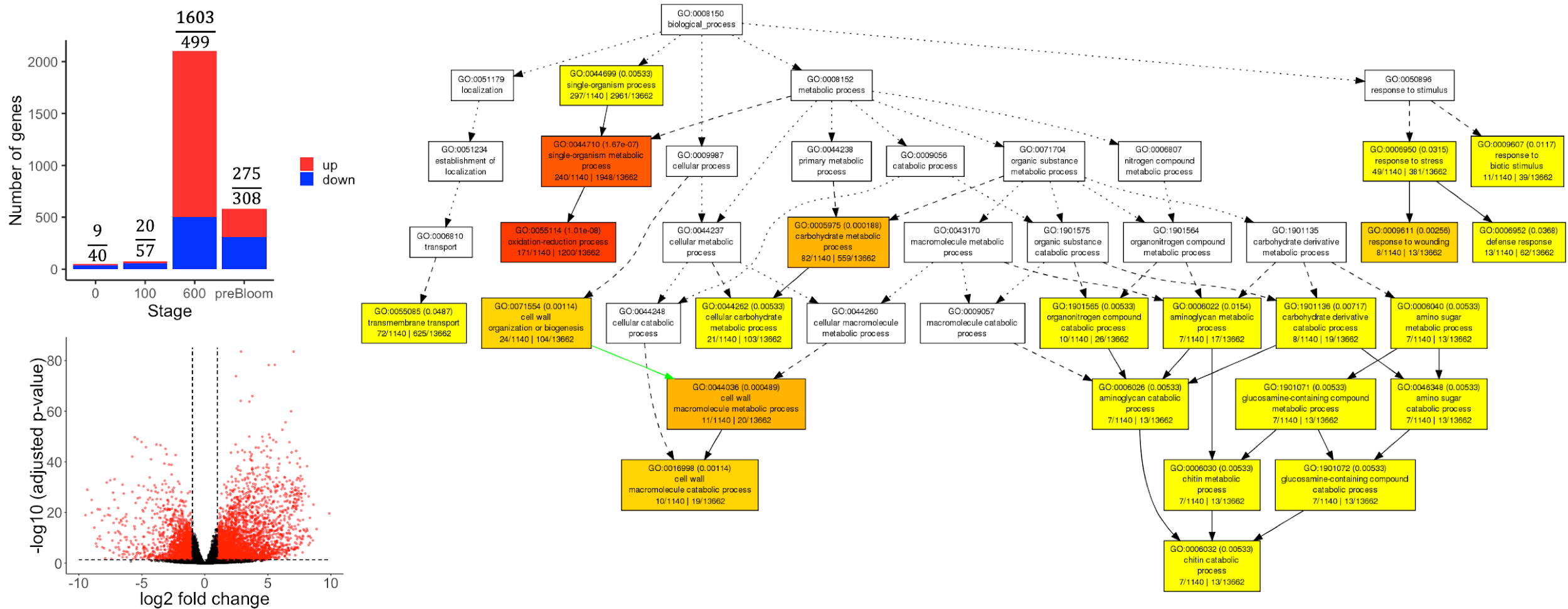


C

A

B

**Supplementary Figure 9**. The differentially expressed genes between peach low chill genotypes and high chill genotypes. (A) The number of DEGs between low chill and high chill genotypes at each time point. (B) Volcano plot of genes differentially expressed at 600 chill hours. Significant genes are in red. (C) The enriched GO terms of the significant DEGs.


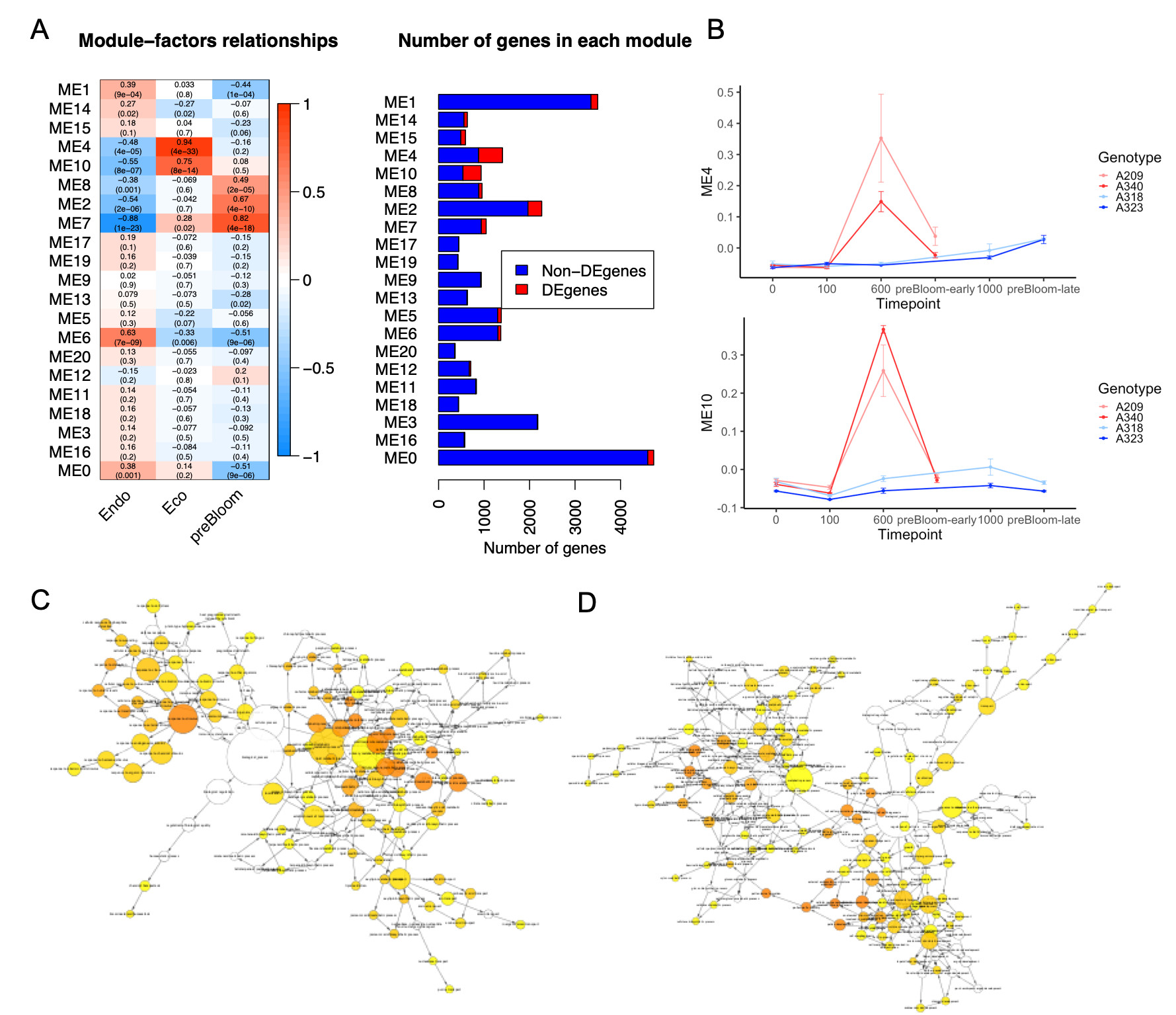


**Supplementary Figure 10**. The co-expression networks identified modules induced at ecodormancy in peach. (A) Left: Module-factor relationships between modules to developmental stages identified by PCA; right: the number of DEGs in each module. (B) The expression profiles of ME4 (top) eigengene and ME10 (bottom) eigengene from dormancy onset to pre-bloom. (C) The enriched GO network of ME4 genes. (D) The enriched GO networks of ME10 genes.


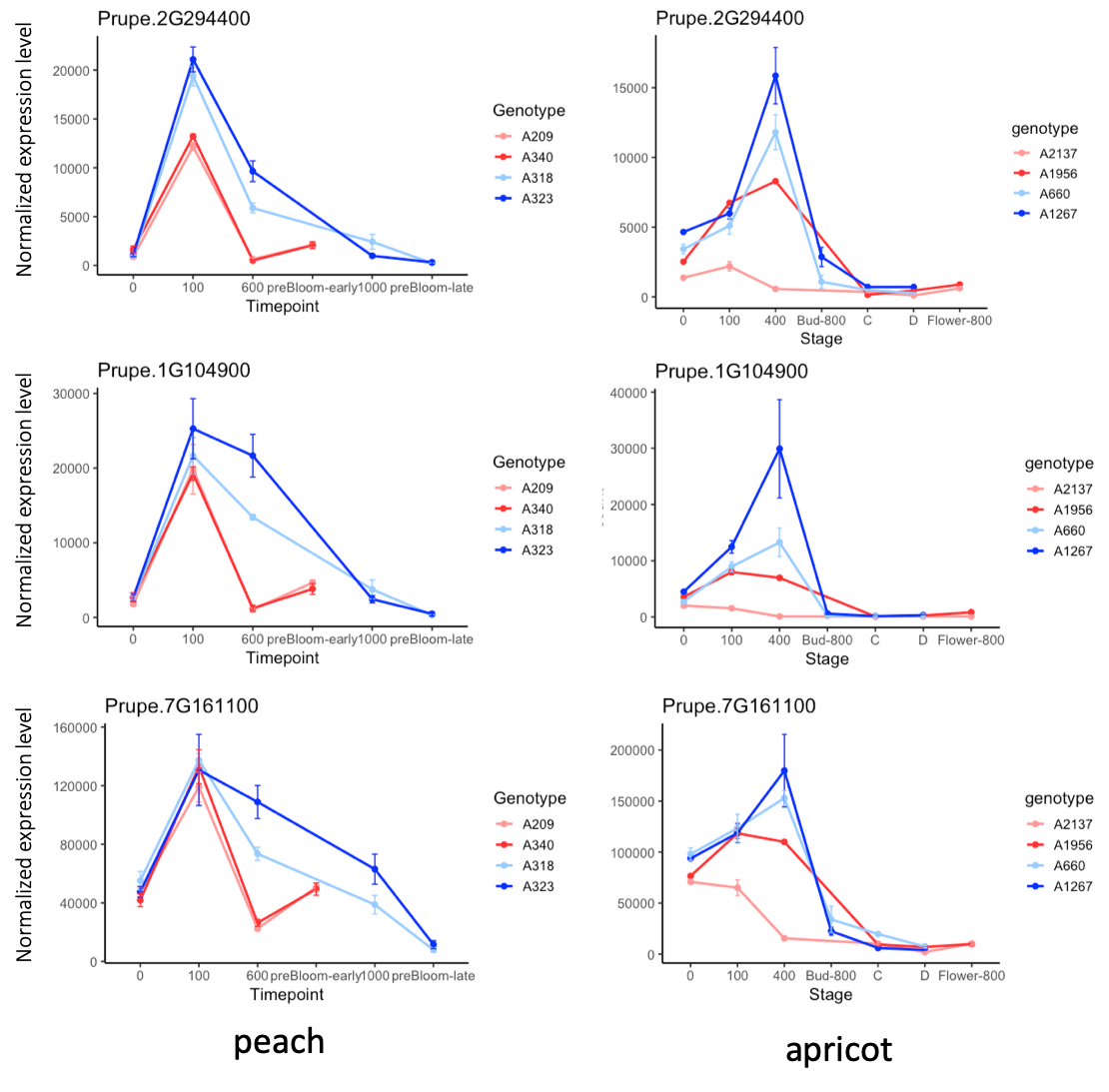


**Supplementary Figure 11**. The expression profiles of cold response genes in peach (left) and apricot (right).


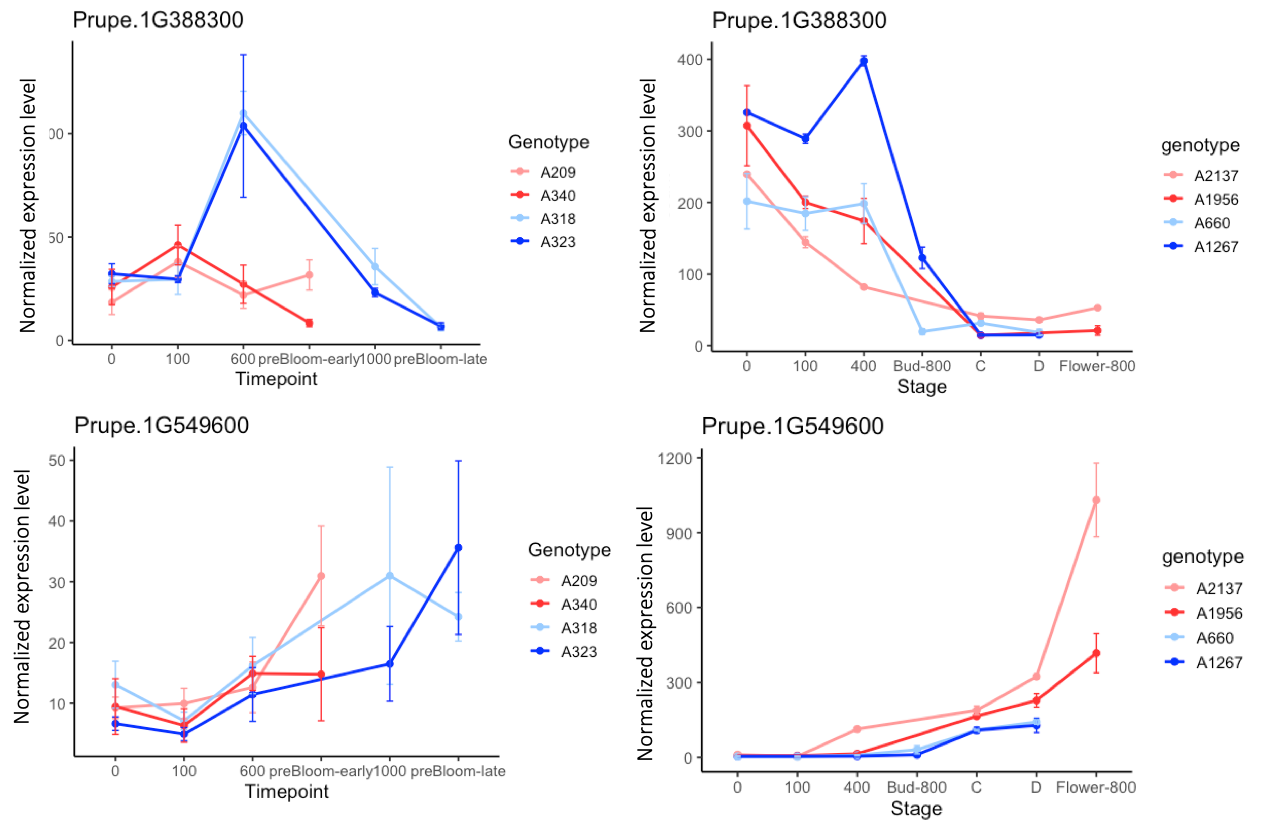


**Supplementary Figure 12**. The expression profiles of pollen related genes in peach (left) and apricot (right).


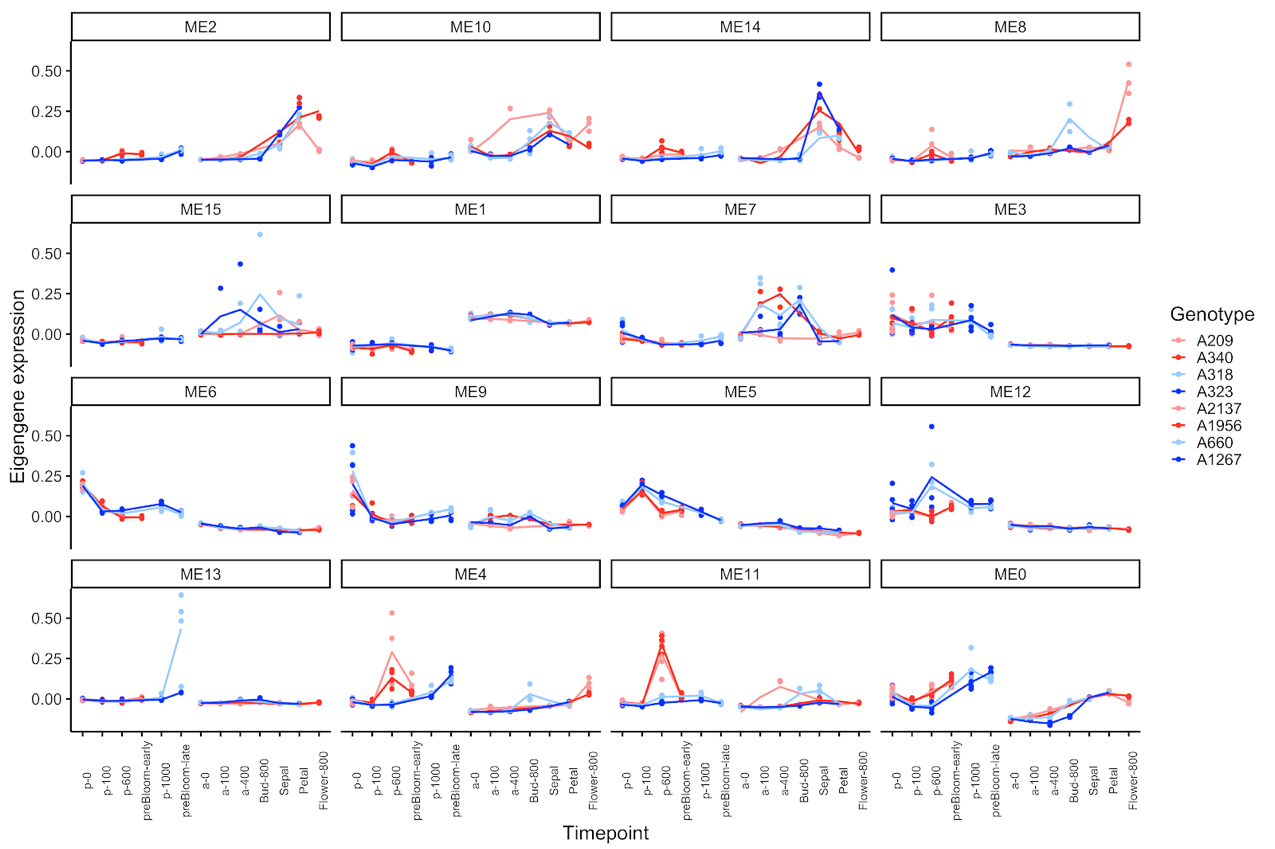


**Supplementary Figure 13**. The expression profiles of co-expression modules identified with the peach and apricot combined dataset. Two red lines represent low chill (early blooming) genotypes, two blue lines represent high chill (late blooming) genotypes. For every subplot, the left panel shows the eigengene expression in peach, while the right panel shows the eigengene expression in apricot.
